# Transcriptomic Analysis Reveals the Positive Role of Abscisic Acid in Endodormancy Maintenance of Leaf Buds of *Magnolia wufengensis*

**DOI:** 10.3389/fpls.2021.742504

**Published:** 2021-11-11

**Authors:** Kunjing Wu, Xiaojing Duan, Zhonglong Zhu, Ziyang Sang, Yutong Zhang, Haiying Li, Zhongkui Jia, Luyi Ma

**Affiliations:** ^1^Beijing Advanced Innovation Center for Tree Breeding by Molecular Design, College of Biological Sciences and Technology, Beijing Forestry University, Beijing, China; ^2^Zhejiang Institute of Subtropical Crops, Zhejiang Academy of Agricultural Sciences, Wenzhou, China; ^3^National Energy R&D Center for Non-food Biomass, Beijing Forestry University, Beijing, China; ^4^Magnolia wufengensis Research Center, Beijing Forestry University, Beijing, China; ^5^Forestry Science Research Institute of Wufeng County, Yichang, China; ^6^College of Forestry, Engineering Technology Research Center of Pinus tabuliformis of National Forestry and Grassland Administration, Beijing Forestry University, Beijing, China

**Keywords:** bud dormancy, RNA-seq, *Magnolia wufengensis*, ABA, dormancy release, cold tolerance

## Abstract

*Magnolia wufengensis* (Magnoliaceae) is a deciduous landscape species, known for its ornamental value with uniquely shaped and coloured tepals. The species has been introduced to many cities in south China, but low temperatures limit the expansion of this species in cold regions. Bud dormancy is critical for plants to survive in cold environments during the winter. In this study, we performed transcriptomic analysis of leaf buds using RNA sequencing and compared their gene expression during endodormancy, endodormancy release, and ecodormancy. A total of 187,406 unigenes were generated with an average length of 621.82 bp (N50 = 895 bp). In the transcriptomic analysis, differentially expressed genes involved in metabolism and signal transduction of hormones especially abscisic acid (ABA) were substantially annotated during dormancy transition. Our results showed that ABA at a concentration of 100 μM promoted dormancy maintenance in buds of *M. wufengensis*. Furthermore, the expression of genes related to ABA biosynthesis, catabolism, and signalling pathway was analysed by qPCR. We found that the expression of *MwCYP707A-1-2* was consistent with ABA content and the dormancy transition phase, indicating that *MwCYP707A-1-2* played a role in endodormancy release. In addition, the upregulation of *MwCBF1* during dormancy release highlighted the enhancement of cold resistance. This study provides new insights into the cold tolerance of *M. wufengensis* in the winter from bud dormancy based on RNA-sequencing and offers fundamental data for further research on breeding improvement of *M. wufengensis*.

## Introduction

Owing to the instability in global climate, many perennial plants have suffered from abnormal weather conditions, including extreme temperatures in winter. Bud dormancy is the temporary suspension of visible growth in plant buds and represents a protective strategy for perennial plants to survive unfavourable climatic changes during winter (Rohde and Bhalerao, 2007). Bud dormancy is traditionally categorised into three phases: paradormancy (PD), inhabited by substances generated from another part of the plant; endodormancy (ED), controlled by internal factors; and ecodormancy (ECD), regulated by the external environment (Lang et al., 1987; Considine and Considine, 2016). Plants cannot resume growth in a favourable environment until ED release (Rohde and Bhalerao, 2007). To break ED, plants need to fulfil chilling requirements (CRs) after accumulating sufficient chilling hours (Arora et al., 2003), as insufficient cold accumulation may delay dormancy release, influence flower morphology, and even impair growth and production (Atkinson et al., 2013). Therefore, it is necessary to evaluate bud dormancy status and assess CRs in perennial trees. Three models are mainly used to calculate CRs in woody perennials: 0–7.2°C model (Weinberger, 1950), Utah model (Richardson, 1974), and dynamic model (Fishman et al., 1987a, b).

Temperature and photoperiod are important environmental signals controlling the seasonal dormancy cycle in perennials (Anderson et al., 2010; Maurya and Bhalerao, 2017). Short photoperiods induce bud formation, bud dormancy induction and apical meristem cessation of shoots (Weiser, 1970; Singh et al., 2017). Moreover, dormancy release requires sufficient chilling accumulation in winter as low temperature mostly regulate dormancy release and bud break (Heide, 2008; Yamane et al., 2011). According to the two different dormancy-related environmental factors, plants can be classified into three types: temperature-sensitive, photoperiod-sensitive, and temperature- and photoperiod-sensitive (Bai et al., 2016).

Phytohormones are a crucial factor influencing bud dormancy in perennials, and endogenous hormones and their balance regulate the induction of and release from dormancy (Sonnewald and Sonnewald, 2014; Liu and Sherif, 2019). Some conventional hormones such as gibberellin (GA), abscisic acid (ABA), and auxin (IAA) participate in the dormancy cycle (Horvath et al., 2003; Vimont et al., 2021). In general, an increase in the ABA content accompanied by a decrease in the GA_3_ and IAA content is observed during the dormant induction phase, whereas the opposite trend is observed during dormancy release in plants (Liu and Sherif, 2019). High levels of IAA and GA_3_ accelerate dormancy release (Rinne et al., 2011; Zhuang et al., 2013) whereas ABA maintains dormancy (Li et al., 2018; Tylewicz et al., 2018). In addition, exogenous ABA application results in a delay in bud break in, for example, *Pyrus pyrifolia* (pear) (Li et al., 2018), *Vitis vinifera* (grape) (Zheng et al., 2015), and *Betula pendula* (birch) (Rinne et al., 1994).

The role of ABA in dormancy has been widely studied at physiological and molecular levels and evidence has indicated that ABA biosynthesis, catabolism, and signalling pathway are involved in the regulation of bud dormancy (Zheng et al., 2018). A rate-limiting enzyme involved in ABA biosynthesis, 9-cis-epoxycarotenoid dioxygenase (NCED), has been indicated to control dormancy at the transcriptional level (Zheng et al., 2015; Li et al., 2018). During catabolism, ABA is degraded by ABA 8’-hydroxylase, which is encoded by cytochrome P450 CYP707A, and the relationship between CYP707A and ABA content has been widely investigated (Cutler and Krochko, 1999; Saito et al., 2004). The ABA signalling pathway consists of two groups of ABA regulators: Protein Phosphatase 2c (PP2Cs) and SNF1-Related Protein Kinase 2 (SnRK2s). Besides, the ABA receptors were identified as Pyrabactin Resistance (PYRs), Pyrabactin Resistance-Like (PYLs), and Regulatory Component of ABA Receptor (RCARs) (Hubbard et al., 2010). ABA binds to PYR/PYL/RCARs and forms PP2C complexes, which inhibit the activity of PP2Cs. PP2Cs can suppress SNF1-related protein kinase 2 (SnRK2s) function via dephosphorylation, which negatively affects ABA signalling, allowing SnRK2s to activate the downstream ABRE-binding factor (AREB/ABF) transcription factors (TFs) (Umezawa et al., 2009; Soon et al., 2011). Several studies have shown that genes related to ABA signalling are involved in dormancy regulation (Yang et al., 2020). In *Hybrid Aspen*, short days induce high levels of ABA which suppresses PICKLE (*PKL*) to induce the expression of *SVP-like* (*SVL*), which is an orthologue of short vegetative phase (*SVP*) and then *SVL* induces callose synthase 1 (*CALS1*) expression to promote the establishment of dormancy (Tylewicz et al., 2018; Singh et al., 2019).

Bud dormancy is an important overwintering process, and many studies have shown that bud dormancy is associated with winter cold resistance at the molecular level. C-repeat binding factor (*CBF*) belongs to the APETALA2/-ETHYLENE RESPONSE FACTOR (AP2/ERF) gene family, regulates many genes related to cold response and tolerance and can be induced by inducer of *CBF* expression (*ICE*) (Chinnusamy et al., 2007; Guo et al., 2018). Dormancy-associated MADS-box (*DAM*)/*SVP*/*SVL* genes are known to control bud dormancy in many species (Singh et al., 2018; Yang et al., 2018; Gao et al., 2021). Therefore, the relationship between *DAM* and *CBF* links between bud dormancy and cold resistance. *PmCBFs* are known to bind to the promoter of *PmDAM6* and activate the expression of *PmCBFs* in *P. mume* (Zhao et al., 2018a, b). In *P. pyrifolia*, the expression of *PpDAMs* is directly induced by the accumulation of CBF by binding to CRT/DRE motifs (Niu et al., 2015; Saito et al., 2015). Li et al. (2019) reported that low temperature induces *PpCBF1-PpDAM2* regulon to function during ED transition (Li et al., 2019). Thus, bud dormancy may be associated with cold tolerance during the winter.

Magnoliaceae plants have high ornamental value and are widely cultivated globally. *Magnolia denudata*, as a common species in north China, has been widely cultivated for its prominent cold tolerance (Yang et al., 2015). *Magnolia wufengensis* (Supplementary Figure 1), a new species of Magnoliaceae, was discovered growing in Wufeng County, Hubei Province, People’s Republic of China (Ma et al., 2006). As a deciduous landscape species with uniquely shaped and colours of the tepals, *M. wufengensis* has been introduced to many cities in south China for its rich biological characteristics and will have a place in global horticultural plants (Shi et al., 2021). However, this is difficult in north China where temperatures can be extremely low, because *M. wufengensis* is more sensitive to the cold and with a deeper dormancy level than other Magnoliaceae species such as *M. denudata* (Yang et al., 2015; Deng et al., 2019; Duan et al., 2019). Bud dormancy is an important biological process that helps plants survive cold temperature in winter. RNA sequencing (RNA-seq) has been recently used to study bud dormancy in many species such as pear (*P. pyrifolia*) (Bai et al., 2013), tea (*Camellia sinensis*) (Hao et al., 2017), sweet cherry (*Prunus avium* L.) (Vimont et al., 2019), and wintersweet (*Chimonanthus praecox*) (Li et al., 2020). In this study, using RNA-seq, we aimed to explore: (i) the cycle period between ED and ECD and the effects of different meteorological factors on dormancy release of *M. wufengensis*, (ii) which key genes and pathways were involved in regulation of different dormancy phases, (iii) the role that hormones, especially ABA, play in endodormancy maintenance, and (iv) the relationship between cold tolerance and different phases of dormancy. This study will provide a foundation for improving cold resistance and thus allowing normal growth in winter and expanding the northern boundary of *M. wufengensis* cultivation.

## Materials and Methods

### Plant Materials

Eight-year-old *M. wufengensis* and *M. denudata* were cultivated in Jiufeng National Forest Park (Beijing, China; 40°3′25″N, 116°6′39″E). The trees were not clipped or chemically treated before sampling. In 2019–2020, one-year shoots with one apical bud were collected from *M. wufengensis* on 2 November (19N_1_), 23 November (19N_2_), 6 December (19D_1_), 14 December (19D_2_), 21 December (19D_3_), 30 December (19D_4_), 12 January (20J_1_), 18 January (20J_2_), and 19 February (20F_1_). In 2020–2021, *M. wufengensis* and *M. denudata* shoots and buds were collected on 5 October (20O_1_), 20 October (20O_2_), 5 November (20N_1_), 20 November (20N_2_), 5 December (20D_1_), 20 December (20D_2_), 5 January (21J_1_), and 20 January (21J_2_). 12 trees were divided into three replicates, and all samples were collected from the same 4 trees at each stage. All leaf bud samples of *M. wufengensis* were stored in liquid nitrogen immediately after collection and then at –80°C until RNA extraction.

### Evaluation of Bud Dormancy Status

The bud dormancy status of leaf buds was evaluated as previously described (Liu et al., 2012) with some modifications. In 2019–2020 and 2020–2021, 1-year-old shoots with one apical bud about 10 cm long (*n* = 7) were sampled and inserted in wet flower mud in a box full of water and allowed to grow in the climate chamber at 25 ± 1.0°C during the day and 22 ± 1.0°C during the night, with a photoperiod of 14 h light/10 h dark and 60% relative humidity. Twenty-one leaf buds grown in three boxes of flower mud were divided into three biological replicates. The water was changed and the base of the shoots was cut every 3–4 days. Dormancy status was determined by the bud break percentage (BBP) after 32 days. We defined the beginning of bud break when the as green leaf tips were enclosing visible leaves. If the buds were at more than 50% bud break after 32 days, then the buds were considered to be released from ED (Yooyongwech et al., 2009).

### Acquisition of Meteorological Data and Chilling Units in *Magnolia wufengensi*s and *Magnolia denudata* During Dormancy

Maximum (T_max_), minimum (T_min_), and average temperatures (T_avg_) were recorded every 15 min using the weather station (WeatherHawk, Campbell Scientific, UT, United States) located in Jiufeng National Forest Park.

Chilling units (CUs) of *M. wufengensis* and *M. denudata* leaf buds were calculated based on 0–7.2°C (Weinberger, 1950) and Utah models (Richardson, 1974).

### Measurements of Phytohormones Contents

The extraction, purification, and determination of endogenous abscisic acid and gibberellin (GA_1_, GA_3_ and GA_4_) were performed using an enzyme-linked immunosorbent assay (ELISA) according to manufacturer’s instructions. The fresh samples (1 g bud) were homogenised in liquid nitrogen and extracted in pre-cold 80% (v/v) methanol with butylated hydroxytoluene (BHT) (1 mmol/L) and kept at 4°C overnight. The samples were centrifuged for 15 min at 5,000 rpm (4°C). Afterward, the extracts were passed through a C18 Sep-Pak Cartridge (Waters, Milford, MA, United States) and dried with N_2_. Then the residues were dissolved in 0.01 mol L^–1^ PBS (pH 7.4) to determine the levels of ABA and GAs content. Calculations of plant hormones by ELISA followed the protocol described in Zhao et al. (2006). The ELISA kits used for the assay were purchased from Saipei Biotechnology Co., Ltd. (Wuhan, China). Each experiment contained three biological and technical replicates.

### RNA Isolation, cDNA Library Construction, and Sequencing

RNA for RNA-seq was isolated separately from the 19N_1_, 19D_1_, and 19D_2_ samples using Plant RNA Purification Reagent for plant tissue according to the manufacturer’s instructions (Invitrogen, Carlsbad, CA, United States) and genomic DNA was removed using DNase I (Takara Bio, Shiga, Japan). Then, RNA quality was determined using the 2100 Bioanalyzer Instrument (Agilent Technologies, Santa Clara, CA, United States) and quantified using NanoDrop 2000 (Thermo Fisher Scientific, Waltham, MA, United States). Only high-quality RNA samples (optical density (OD)260/280 = ∼1.8–2.2, OD260/230 ≥ 2.0, RNA integrity number ≥ 6.5, 28S:18S ≥ 1.0, quantity > 1 μg) were used to construct the sequencing library.

RNA purification, reverse transcription, library construction, and sequencing were performed at Shanghai Majorbio Bio-pharm Biotechnology Co., Ltd. (Shanghai, China) according to the manufacturer’s instructions (Illumina, San Diego, CA, United States). The RNA-seq transcriptome libraries of *M. wufengensis* were prepared using TruSeq RNA Sample Prep Kit (Illumina). Poly(A) mRNA was purified from total RNA using oligo-dT-attached magnetic beads (Invitrogen) and then fragmented using the fragmentation buffer. Using these short fragments as templates, double-stranded complementary DNA (cDNA) was synthesised using SuperScript Double-Stranded cDNA Synthesis Kit (Invitrogen) with random hexamer primers (Illumina). Subsequently, the synthesised cDNA was subjected to end-repair, phosphorylation, and “A” base addition according to Illumina’s library construction protocol. Libraries were selected for size using cDNA target fragments of 200–300 bp on 2% Low Range Ultra Agarose (Bio-Rad) followed by PCR amplification using Phusion DNA polymerase (New England Biolabs, Boston, MA, United States) for 15 PCR cycles. After quantification using TBS380, two RNA-seq libraries were sequenced in a single lane on NovaSeq 6000 Sequencing System (Illumina) for 2 × 150 bp paired-end reads. Each experiment included three biological replicates.

### *De novo* Assembly and Sequence Annotation

The raw paired-end reads were trimmed and quality controlled using SeqPrep^1^ and Sickle^2^ with default parameters. Subsequently, clean data from *M. wufengensis* were used to perform *de novo* assembly with Trinity^3^ (Grabherr et al., 2011). All the assembled transcripts were searched against the National Center for Biotechnology Information protein non-redundant (NR), Clusters of Orthologous Genes (COG), and Kyoto Encyclopedia of Genes and Genomes (KEGG) databases using BLASTX to identify the proteins that had the highest sequence similarity with the given transcripts to retrieve their function annotations and typical cut-off E-values were set as less than 1.0 × 10^––5^. Blast2GO^4^ (Conesa et al., 2005) programme was used to obtain gene ontology (GO) annotations of uniquely assembled transcripts for describing their biological processes, molecular functions, and cellular components. Metabolic pathway analysis was performed using KEGG^5^ (Ogata et al., 1999).

### Differential Expression Analysis and Functional Enrichment

To identify differentially expressed genes (DEGs) between two different samples, the expression level of each transcript was calculated according to the transcripts per million reads method. RSEM^6^ (Li and Dewey, 2011) was used to quantify gene abundance. DEG analysis was performed using DESeq2 (Love et al., 2014) and EdgeR (Robinson et al., 2009) with DEGs |log2FC| > 1 and Q value ≤ 0.05 (DESeq2 or EdgeR) considered to be significant. In addition, functional enrichment analysis using GO and KEGG were performed to identify which DEGs were significantly enriched in GO terms and metabolic pathways with a Bonferroni-corrected *p* value ≤ 0.05 compared to the whole-transcriptome background. GO functional enrichment and KEGG pathway analyses were carried out using Goatools^7^ and KOBAS^8^ (Xie et al., 2011).

Venn diagrams were drawn and trend analysis was performed using Venny 2.1^9^ and Short Time-series Expression Miner software (STEM) (Ernst and Bar-Joseph, 2006), respectively.

### Exogenous Abscisic Acid Treatment

For ABA treatment, nine shoots of *M. wufengensis* were collected from the 12 trees from November 2020 to January 2021 and sprayed with 100, 200, or 300 μM ABA (Aidlab, Beijing, China) and 0.2% ethanol (mock treatment) at approximately 13:00 for three consecutive days. Each treatment was executed with three biological and technical replicates. Buds were collected at 7, 14, 21, and 28 days after ABA treatment and stored immediately in liquid nitrogen and then at –80°C. Other shoots that grew in the chamber environment mentioned above for 32 days were used to measure BBP.

### Identification and Validation of Cold-Related Genes by Cold Acclimation

To ensure DEGs identified by RNA-seq involve in cold tolerance of *M. wufengensis*, a cold acclimation experiment was conducted to valid their functions. Three apical buds were selected randomly and used for experiments during the whole cold acclimation treatment in September 2017 (autumn). The experiment included three temperatures for analysis. A room temperature of 22°C in a low-temperature incubator (3M, United States) served as the control. The samples in other groups of *M. wufengensis* buds were treated for seven days sequentially in low-temperatures incubators at the following two different experimental temperatures: low temperature of 12 and 4°C. Two days were left for cooling slowly in temperatures incubator between two groups (Supplementary Figure 2).

### Quantitative PCR Analysis of Gene Expression

RNA of *M. wufengensis* buds for quantitative PCR (qPCR) was extracted using the HiPure HP Plant RNA Mini Kit (Magen, Shanghai, China) according to the manufacturer’s instructions and genomic DNA was removed using DNase I. cDNA used for qPCR was reverse transcribed from 2 μg of purified RNA in a 20 μL reaction volume based on the manufacturer’s instructions (G592, Applied Biological Materials, Richmond, BC, Canada). The qPCR primers were designed using Beacon Designer 7 (PREMIER Biosoft International, Palo Alto, CA, United States) and passed the specificity test. qPCR was carried out on a StepOnePlus Real-Time PCR System (Applied Biosystems, United States) with a final reaction volume of 10 μL containing 5 μL TB Green Premix Ex Taq (Tli RNaseH Plus; Takara Bio) (2X), 0.2 μL each of ROX Reference Dye (50X) (Takara Bio), upstream primer, and downstream primer, 1.0 μL cDNA, and 3.4 μL double-distilled water. *MwACTIN* was used for a reference gene for analysis. The primer sequences used in qPCR are listed in Supplementary Table 1. Each sample included three biological and technical replicates.

### Statistical Analysis

The study was conducted with a completely randomised design. The data were analysed using one-way analysis of variance followed by least significant difference test and *p* value <0.05 was considered significant. Graphs were constructed using SigmaPlot version 10 (Systat Software, San Jose, CA, United States) and R Project (R Foundation for Statistical Computing, Vienna, Austria). All data were analysed using SPSS Statistics version 20 (IBM, Armonk, NY, United States).

## Results

### Dormancy Status and Chilling Requirement of Buds in *Magnolia wufengensis* and *Magnolia denudata* During Natural Overwintering

**FIGURE 1:**
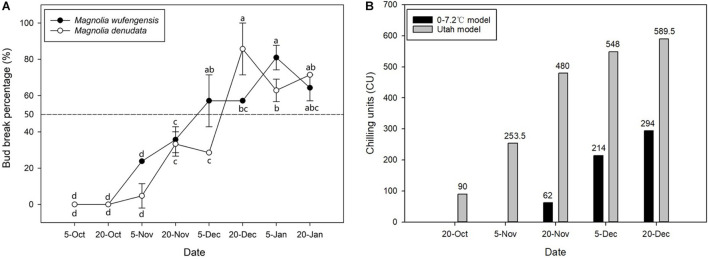
Bud break percentage and chilling units of *Magnolia wufengensis* and Magnolia denudata during 2020–2021 dormancy phase under natural conditions. **(A)** Bud break percentage of *Magnolia wufengensis* and Magnolia denudata was assessed after 32 days in 2020–2021. **(B)** Chilling units were calculated based on 0–7.2°C and Utah models beginning on 14 November and 29 September 2020, respectively. Each experiment was performed with three biological replicates. Different letters above bars indicate a significant difference among bud break percentage according to ANOVA and LSD test (*p* < 0.05).

To study the relationships between bud dormancy and cold tolerance, it is imperative to define the status of bud dormancy. As is shown in Figure 1A, no bud breaking was observed in *M. wufengensis* and *M. denudata* on 20O_2_; however, BBP increased with progress in chilling accumulation mainly in November and December. In *M. wufengensis*, the apical leaf buds were determined in the ED phase before 20N_2_ and ED release between 20N_2_ and 20D_1_, when the number of CUs reached 62–214 CUs and 480–548 CUs based on the 0–7.2°C and Utah models, respectively. In addition, the ED release occurred between 5 December and 20 December in *M. denudata*, which was later than that in *M. wufengensis* with CUs reaching 214–294 CUs and 548–589.5 CUs based on the 0–7.2°C and Utah models, respectively (Figure 1B).

### Phytohormone Concentration During Bud Dormancy of *Magnolia wufengensis*

**FIGURE 2:**
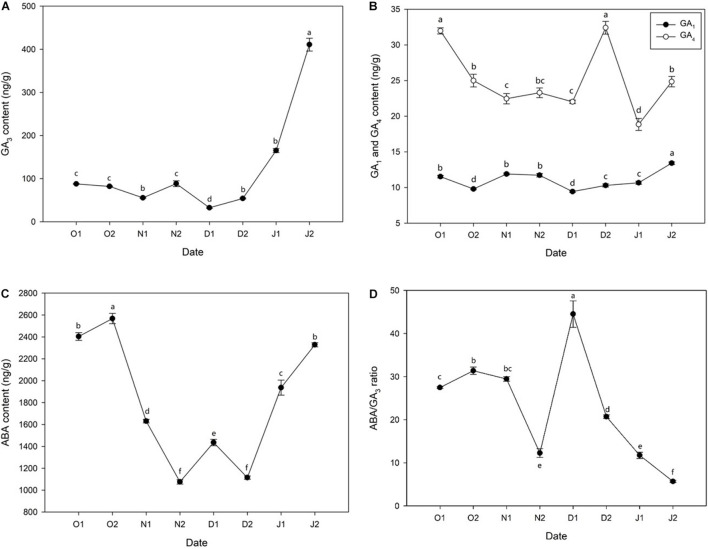
Concentration of phytohormones [**(A)** gibberellin 3 (GA3), **(B)** gibberellin 1 (GA1) and gibberellin 4 (GA4), **(C)** abscisic acid (ABA), **(D)** ABA/GA3 ratio] in *Magnolia wufengensis* during the season transition in 2020–2021. Each experiment was performed with three biological replicates. Different letters above bars indicate a significant difference among hormones according to ANOVA and LSD test (*p* < 0.05).

GA_3_ content was low before 21J_1_ and peaked at 21J_2_, and a small peak appeared at 20N_2_ (before ED release) (Figure 2A) and a similar result was observed regarding the content of GA_1_ (Figure 2B). In addition, the content of GA_4_ decreased during ED release phase and a peak at 20D_2_ was observed (Figure 2B). Moreover, before ED release, the concentration of ABA kept increasing until at 20O_2_ and then decreased rapidly to the lowest level at 20N_2_ with dormancy release, with a slight increase at 20D_1_ and considerable reduction immediately at 20D_2_. After ED release, ABA levels sharply increased after 20D_2_ and increased further at 21J_2_ (Figure 2C). In addition, the ratio of content of ABA/GA_3_ increased before ED release and experienced a sharp decrease after ED release (Figure 2D).

### Transcriptome Sequencing, *de novo* Assembly, and Annotation of *Magnolia wufengensis* Unigenes During Bud Dormancy

Three libraries 19N_1_ (ED, control, BBP = 0%), 19D_1_ (ED release phase, 0% < BBP < 50%), and 19D_2_ (ECD, BBP > 50%) (Supplementary Figure 3) were constructed from cDNA obtained from more than three apical buds and sequenced on the NovaSeq 6000 platform. Approximately 59.75 GB of clean reads was obtained after quality control, and Q30 percentage and guanine and cytosine content (GC) percentage were more than 92.14 and 46.92% in the nine samples, respectively (Table 1). The *de novo* assembly using Trinity yielded 187,406 unigenes ranging from 201 bp to 14,669 bp with an average length of 621.82 bp, and N50 of 895 bp (Table 2). In general, the number of unigenes decreased with the increase in gene length, and the largest proportion of unigenes was between 200 bp and 500 bp (123,562, 66%), followed by 501 bp to 1,000 bp (35,770, 19%), and 1,001 bp to 1,500 bp (11,895, 6%) (Supplementary Figure 4A).

**TABLE 1:** Sequencing the *Magnolia wufengensis* transcriptome from nine apical leaf samples from plants from endodormancy (ED; 19N_1__1, 19N_1__2, 19N_1__3), before ED release (19D_1__1, 19D_1__2, 19D_1__3), and ecodormancy (ECD; 19D_2__1, 19D_2__2, 19D_2__3).

Sample	Clean reads	Clean bases	Error rate (%)	Q20 (%)	Q30 (%)	GC content (%)
19N1_1	43633612	6.46E + 09	0.0272	97.16	92.14	47.05
19N1_2	43329432	6.42E + 09	0.026	97.61	93.15	47.14
19N1_3	41462488	6.16E + 09	0.0258	97.7	93.35	47.09
19D1_1	43446866	6.44E + 09	0.026	97.61	93.18	46.92
19D1_2	47754902	7.1E + 09	0.026	97.61	93.17	46.96
19D1_3	44602650	6.52E + 09	0.0268	97.29	92.43	46.93
19D2_1	43032290	6.4E + 09	0.0263	97.49	92.94	49.52
19D2_2	46841758	6.93E + 09	0.0261	97.58	93.16	49
19D2_3	49290604	7.31E + 09	0.0258	97.7	93.39	49.91

**TABLE 2:** Statistical results of transcriptome unigenes.

Total number	200–500 bp	501–1,000 bp	1,001–1,500 bp	>1,500 bp	N50	Max length	Min length	Average length
187,406	123,562	35,770	11,895	16,179	895	14,669	201	621.82

The assembled unigenes were compared against NR, Swiss-Prot, Pfam, COG, GO, and KEGG databases using BLASTX (E-value < 1e^–5^). Simultaneously, we found that the number of unigenes successfully annotated to the NR database was the highest (78,142; 41.7%), followed by COG (71,437; 38.1%), KEGG (61,840; 33.0%), Pfam (55,774; 29.8%), Swiss-Prot (55,692; 29.7%), and GO (43,500; 23.2%) (Supplementary Figure 4B). With respect to species, the unigene sequences were most similar to genes from *Quercus suber* (29,500), *Cinnamomum micranthum* (11,576), *Carpinus fangiana* (5,871), *Nelumbo nucifera* (3,592), and *V. vinifera* (3,052) using BLASTX matches (Supplementary Figure 4C).

KEGG analysis revealed 61,840 unigenes to be significantly mapped to 139 KEGG pathways and classified into six categories: Metabolism, Genetic Information Processing, Environmental Information Processing, Cellular Processes, Organismal Systems, and Human Diseases. The highest unigenes representation pathways in “Metabolism” were carbohydrate metabolism (4,090) and amino acid metabolism (2,911), those in “Genetic Information Processing” were translation (4,895) and folding, sorting, and degradation (3,342). Signal transduction (823), transport and catabolism (2,138), environmental adaptation (852), and endocrine and metabolic disease (116) were most associated with “Environmental Information Processing,” “Cellular Processes,” “Organismal Systems,” and “Human Diseases,” respectively (Supplementary Figure 4D).

Based on GO analysis, 43,500 unigenes were classified into three main categories: biological process, cellular component, and molecular function. Biological process was mainly comprised of proteins involved in cellular process (29,572), metabolic process (27,167), and biological regulation (8,340). The most represented cellular components were cell part (29,063), membrane part (22,285), and organelle (16,681). In addition, we found a high number of unigenes involved in binding (36,757), catalytic activity (35,199), and transporter activity (5,244) in molecular function (Supplementary Figure 4E).

### Changes in Gene Expression, Gene Expression Patterns, and Enrichment Analysis of Differentially Expressed Genes During Bud Dormancy

**FIGURE 3:**
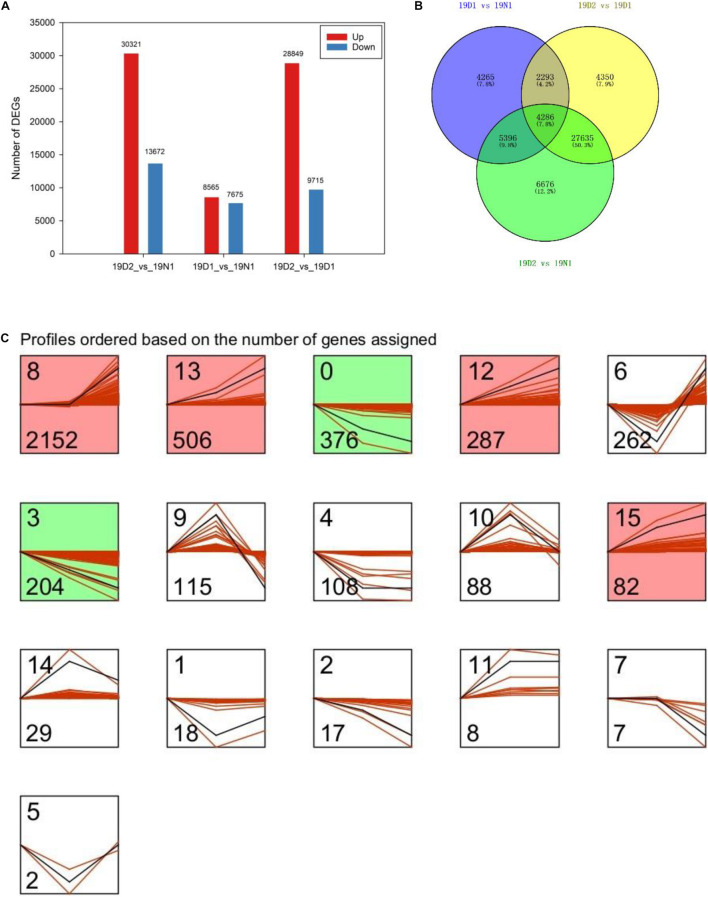
Expression patterns of differentially expressed genes (DEGs) during different dormancy phases in *Magnolia wufengensis*. **(A)** Changes in gene expression profile during different dormancy phases. **(B)** Venn diagram of DEGs between 19D1 versus 19N1, 19D2 versus 19N1, and 19D2 versus 19D1. **(C)** Trend analysis of 4,286 intersecting DEGs in panel **(B)**.

Unigenes with *p* value < 0.05 or |log2FC| ≥ 1 were defined as DEGs. Among the DEGs, 8,565 and 30,321 were upregulated and 7,675 and 13,672 genes were downregulated at 19D_1_ and 19D_2_, respectively. Moreover, 28,849 upregulated and 9,715 downregulated unigenes were identified between 19D_2_ and 19D_1_. The number of DEGs of 19D_2_ versus 19N_1_ were the highest, followed by 19D_2_ versus 19D_1_ and 19D_1_ versus 19N_1_ (Figure 3A). To further explore DEGs related to dormancy release under natural conditions, a Venn diagram was drawn between 19D_1_ versus 19N_1_, 19D_2_ versus 19N_1_, and 19D_2_ versus 19D_1_, and 4,286 DEGs were found to intersect all three groups (Figure 3B). To distinguish the changing patterns in gene expression, gene expression profile clustering was performed. From this, 4,286 genes were assigned to 16 different profiles by STEM and six profiles that were significantly enriched from 19N_1_ to 19D_2_ were identified (Figure 3C).

Compared to 19N_1_, GO analysis of DEGs at 19D_1_ demonstrated that genes related to membrane structure and transcription were overexpressed. Biological process, cellular component, and molecular function, “single-organism transport,” “cellular component,” and “oxidoreductase activity” were the most enriched GO categories. At 19D_2_, in biological process, the major subcategories were “metabolic process” and “single-organism process.” In cellular component, “cellular component,” “cell part,” and “intracellular part” were the most representative subcategories, and “oxidoreductase activity,” “transporter activity,” and “RNA binding” were the top three subcategories compared to 19N_1_.

Compared to 19N_1_, KEGG pathway enrichment analysis for DEGs indicated that four pathways – “ribosome (map03010),” “oxidative phosphorylation (map00190),” “plant-pathogen interaction (map04626)” and “plant hormone signal transduction (map04075)” were significantly enriched at 19D_1_. At 19D_2_ versus 19N_1_, “spliceosome (map03040)” followed by “oxidative phosphorylation (map00190),” and “plant hormone signal transduction (map04075)” were significantly enriched. Compared with D_1_, “ribosome (map03010),” “oxidative phosphorylation (map00190),” and “spliceosome (map03040),” were significantly enriched at 19D_2_ (Supplementary Table 2).

### Hormone Signal Transduction Related Genes Were Expressed During Bud Dormancy Transition

**FIGURE 4:**
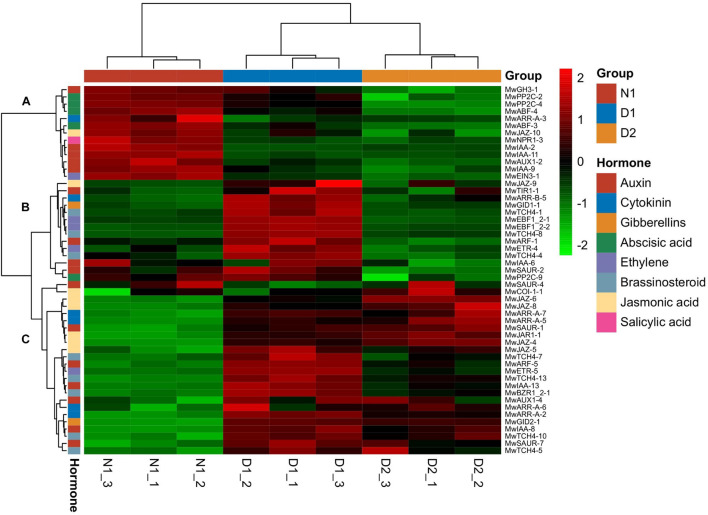
Heatmap of differentially expressed genes (DEGs) involved in plant hormone signal transduction (map04075) during different dormancy phases at 2019–2020 in *Magnolia wufengensis*.

Based on KEGG annotation, DEGs related to phytohormones play an important role in dormancy transition. A total of 51 DEGs related to plant hormone signal transduction (Supplementary Table 3) divided into three main gene clusters as shown in Figure 4. Cluster A (13 genes) was highly expressed at the 19N_1_ (ED) stage, and showed low expression levels during dormancy release, which indicated that the genes in this cluster may be involved in breaking ED. Among the DEGs in cluster A, two genes involved in ABA signalling, *PP2C* and *ABF*, and five auxin-related genes, including three *IAA*, and one each of *GH3* and *AUX*, were found, which indicates that ABA and auxin signalling were activated during dormancy release. Cluster B contained 14 genes that showed low expression level at N_1_, and sharply increased at 19D_1_before decreasing to a relatively low level at 19D_2_. Among them, DEGs associated with auxin (*IAA, SAUR, ARF*, and *T1R1*), ethylene (*EBF* and *ETR*), and brassinosteroid (*TCH4*) regulation were most abundant in this expression profile cluster. Cluster C, with the largest number of DEGs (24 genes), exhibited a low expression level at 19N_1_, which gradually increased at 19D_1_ and part of 19D_2_. Among them, DEGs that responded to jasmonic acid (*COI1, JAZ*, and *JAR1*), cytokinin (*ARR-A*), auxin (*SAUR, ARF, IAA* and *AUX1*), and brassinosteroid (*TCH4* and *BZR1*) exhibited a similar expression pattern to that in cluster C.

### Transcription Factors Were Active During Endodormancy Release

A total of 1,001 TF genes (552 upregulated and 449 downregulated) that were active during dormancy transition were identified. These TFs were mainly concentrated in MYB_superfamily, C2H2, C2C2, bHLH, bZIP, AP2/ERF, NAC, and WRKY. Among all the evaluated genes, the number of genes of the MYB_superfamily was the highest with 131 genes (59 upregulated and 72 downregulated), followed by C2H2 with 103 genes (90 upregulated and 13 downregulated), C2C2 with 93 genes (56 upregulated and 37 downregulated), bHLH with 86 genes (45 upregulated and 41 downregulated), bZIP with 80 genes (64 upregulated and 16 downregulated), AP2/ERF with 66 genes (35 upregulated and 31 downregulated), NAC with 55 genes (25 upregulated and 30 downregulated), and WRKY with 48 genes (26 upregulated and 22 downregulated) (Supplementary Table 4).

### Validation of RNA-Seq Results Using Quantitative PCR

Ten DEGs were randomly selected to demonstrate the reliability of RNA-seq using qPCR. The trends of genes during different dormancy phases using qPCR were consistent with the RNA-seq results, indicating favourable reliability of RNA-seq (Supplementary Figure 5).

### Abscisic Acid at a Concentration of 100 μM Promoted Endodormancy Maintenance in *Magnolia wufengensis*

**FIGURE 5:**
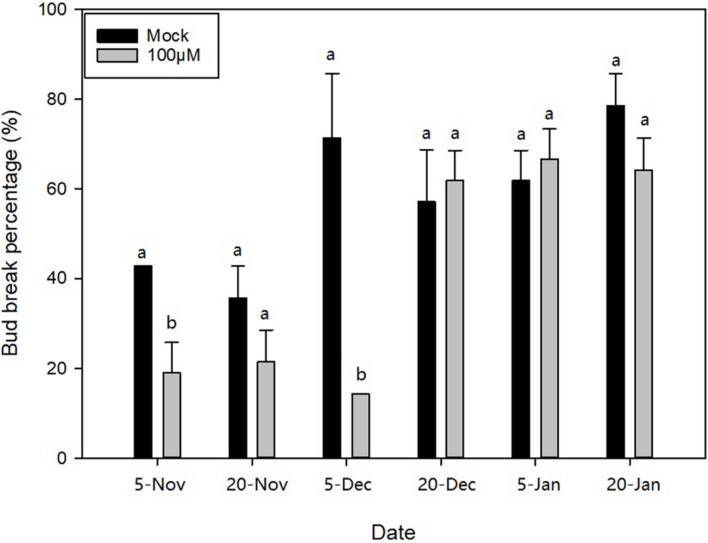
Bud break percentage after abscisic acid (ABA) treatment during endodormancy (ED) to ecodormancy (ECD) during 2020–2021 in *Magnolia wufengensis*. Each experiment was performed with three biological replicates. Different letters above bars indicate a significant difference among bud break percentage under different concentration of ABA according to ANOVA and LSD test (*p* < 0.05).

Based on the transcriptional analysis, we analysed and inferred that hormone metabolism, signal transduction, and especially ABA may play important roles in dormancy transition. Therefore, to further figure out the function of ABA with respect to ED release, three different exogenous concentrations of ABA (100, 200, and 300 μM) were applied to apical buds on 20N_1_, 20N_2_, 20D_1_, 20D_2_, 21J_1_, and 21J_2_ in *M. wufengensis*, and their BBP with exogenous ABA and mock treatments (0.2% ethanol) was compared after 32 days. Based on the evaluated dormancy status, 20N_1_ and 20N_2_ were in ED and ready to release from ED, and 20D_1_, 20D_2_, 21J_1_, and 21J_2_ were in ECD. BBP on 20N_1_ and 20D_1_ significantly decreased after treatment with 100 μM ABA (Figure 5), whereas ABA at concentration had almost no effect on the germination rate in ECD (20D_2_, 21J_1_ and 21J_2_). This implies that during ED or the ED release phase, ABA at a concentration of 100 μM played a positive role in ED maintenance and was therefore selected for further investigation.

### Identification and Expression of Abscisic Acid-Related Genes During Dormancy Transition Under Natural Environment

**FIGURE 6:**
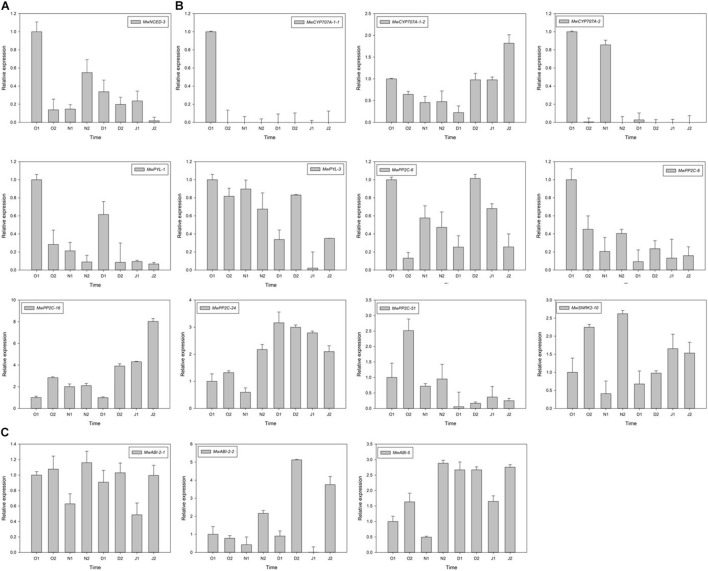
Relative gene expression levels of differentially expressed genes (DEGs) involved in abscisic acid (ABA) biosynthesis **(A)**, metabolism **(B)**, and signal transduction **(C)** under natural condition during 2020–2021 in *Magnolia wufengensis*. Each experiment was performed with three biological replicates. Each bar represents the mean ± SEM of three biological replicates.

To further study the molecular mechanism of action of ABA on ED release, one *NCED*, three *CYP707A*, two *PYL*, five *PP2C*, one *SNRK2*, and three *ABI* DEGs related to ABA synthesis, metabolism, and signalling were identified using RNA-seq. Among these genes, the expression of *MwNCED-3* was downregulated and showed a low expression before dormancy release, and then increased during dormancy release (Figure 6A). The expression of *MwCYP707A-1-2* declined toward ED release and steadily increased after ED release, which is consistent with the content of ABA, whereas almost no expression of *MwCYP707A-1-1* and *MwCYP707A-2* was observed during the entire dormancy release phase (Figure 6B). Moreover, the expression patterns of genes related to ABA signalling were also determined. The expression levels of *MwPYL-1/3* genes decreased before dormancy release and increased rapidly during dormancy release, and then decreased steadily at ECD. The same expression pattern was observed in *MwPP2C-6*. On the contrary, *MwSNRK2-10* slowly increased before dormancy release, peaked on 20N_2_, decreased rapidly during dormancy release, and increased thereafter at ECD. In addition, *MwPP2C-24* and *MwABI-5* increased before dormancy release and dropped with ECD development (Figure 6C).

### Expression of C-Repeat Binding Factor and Inducer of C-Repeat Binding Factor Expression Genes During Dormancy Transition Under Natural Conditions

**FIGURE 7:**
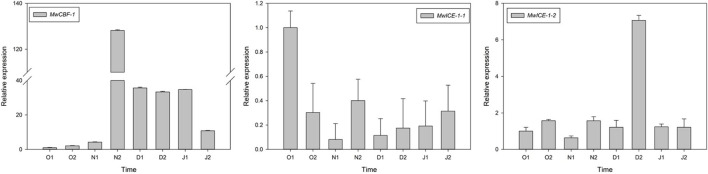
Relative gene expression levels of differentially expressed genes (DEGs) involved in cold resistance under natural condition during 2020–2021 in *Magnolia wufengensis*. Each experiment was performed with three biological replicates. Each bar represents the mean ± SEM of three biological replicates.

To understand the relationship between dormancy transition and cold tolerance in winter, we identified one *CBF1* and two *ICE1* genes in our transcriptome data and measured their expression patterns during the natural ED process in *M. wufengensis*. To confirm their functions on cold resistance, we conducted an experiment under cold acclimation and found that *MwCBF-1* and *MwICE-1-1* were induced under cold stress, indicating that the two genes were associated with cold tolerance (Supplementary Figure 6). Among these genes, the expression of *MwCBF-1* was upregulated to 20N_2_ and showed a high expression before dormancy release, and then decreased but still kept a high expression after ED release. The expression of *MwICE-1-1* decreased to N_1_ and then increased slowly at ECD. The expression level of *MwICE-1-2* showed a high expression at 20D_2_ but maintained a relatively low expression at other phases (Figure 7).

### Expression Analysis of Genes Related to Abscisic Acid and Cold Tolerance in Response to Exogenous Abscisic Acid Treatment

**FIGURE 8:**
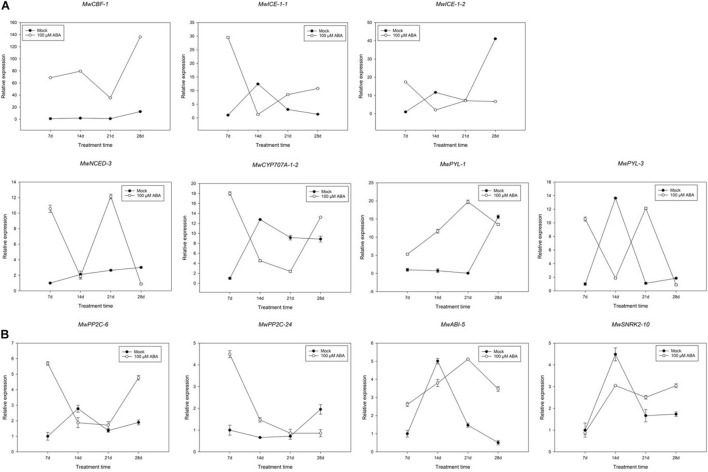
Relative gene expression levels of specific differentially expressed genes (DEGs) involved in abscisic acid (ABA) **(A)** and cold resistance **(B)** under ABA treatment during 2020–2021 in *Magnolia wufengensis*. Each experiment was performed with three biological replicates. Each bar represents the mean ± SEM of three biological replicates.

The significantly decreased BBP indicated that ABA promoted ED maintenance. To further determine the function of ABA in the maintenance of ED, the responses of buds collected at 20D_1_ were compared between mock and ABA treatment group. Genes related to dormancy transition under natural conditions were focussed on (Figure 8). The expression of *MwCBF-1* induced by ABA was considerably upregulated compared to that in the mock treatment and a similar increase was observed in *MwPYL-1* and *MwABI-5*. Fluctuations were observed in the expression of *MwNCED-3* and *MwPYL-3* during the whole treatment time. In addition, a slight decreasing trend can be seen in the expression of the two *PP2C* genes in the previous 21 days, while there was a significant increase in *MwPP2C-6* on the 28th day after treatment which was the opposite to the trend observed for *MwSnRK-2-10*. The expression of *MwCYP707A-1-2* was similar to that of *MwPP2C-6*, showing a high expression at the 7th and 28th days and maintaining a relatively low expression at the 14th and 21st days after ABA treatment.

### Expression Analysis of Genes Related to Bud Dormancy in Response to Exogenous Abscisic Acid Treatment

**FIGURE 9:**
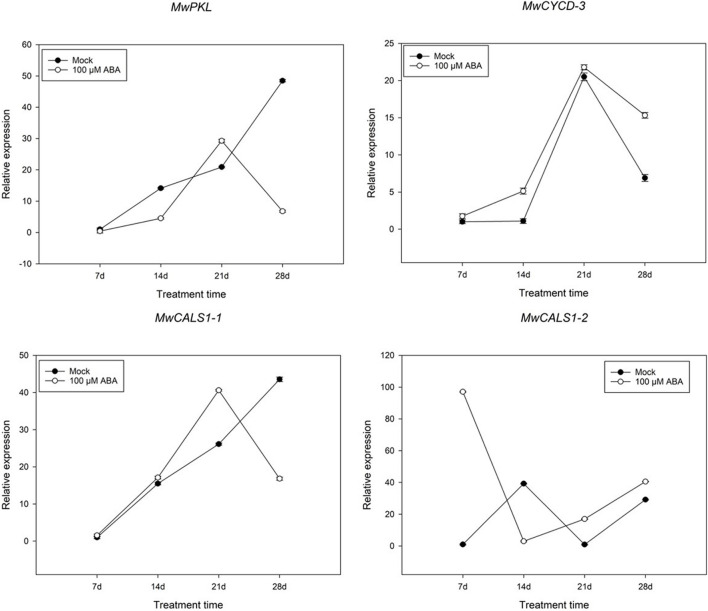
Relative gene expression levels of specific differentially expressed genes (DEGs) involved in bud dormancy under ABA treatment during 2020–2021 in *Magnolia wufengensis*. Each experiment was performed with three biological replicates. Each bar represents the mean ± SEM of three biological replicates.

In addition to analysing the expression pattern of ABA and cold-related genes, several genes such as D-type cyclin (*CYCD*), *PKL* and *CALS1* involving in bud dormancy have also been identified. Among the genes, one *CYCD3*, one *PKL* and two *CALS1* genes were differently expressed during dormancy transition. To further study whether a similar model induced by ABA exists in *M. wufengensis*, we measured the expression of the dormancy-related genes under ABA treatment (Figure 9). The expression of *MwCYCD-3* increased in the previous 21 days and was considerably promoted by ABA and a similar trend exists in *MwCALS-1-1* in the first 21 days. In addition, the expression of *MwPKL* was depressed by ABA throughout the treatment time except the 21st day. During dormancy release, the expression *MwCYCD3* and *MwCALS-1-1/2* were promoted by ABA, but *MwPKL* expression was depressed by ABA.

## Discussion

### Chilling Requirement and Environmental Factors Affected Bud Dormancy

The 0–7.2°C and Utah models are widely used to assess CRs in perennial trees (Yang et al., 2021). As shown in Figure 1B, temperature steadily dropped to 7.2°C relatively late, so no CUs were accumulated before 20N_1_ under the 0–7.2°C model. The Utah model is often used for cold regions such as north China, as it takes into consideration the accumulation effect of different temperatures (Erez et al., 1990). Our results were thus consistent with those of previous studies. Based on the Utah model, *M. wufengensis* underwent lesser CR to break dormancy than *M. denudata*. In addition, we found that the lower the temperature, the higher the BBP. Based on our results, the buds accumulated enough CUs to break ED before 20D_1_.

### Transcriptome Data Revealed Phytohormones Involved in Bud Dormancy Release

Bud dormancy is associated with phytohormones in many species (Cooke et al., 2012). GA and ABA regulate dormancy induction and release (Yue et al., 2018; Yang et al., 2019). In general, ABA levels increase with the establishment of dormancy (PD to ED) and decrease during dormancy release (ED to ECD) while GA_3_ exhibits opposite trends during dormancy transition. For example, in wintersweet, the content of ABA increases with the chilling accumulation from PD to ED and then decreases after dormancy release, and that of GA_3_ decreases with the length of dormancy (Li et al., 2020). Similar results have been observed in many other perennial species such as pear (*P. pyrifolia*) (Tuan et al., 2017; Ito et al., 2021), peach (*Prunus persica*) (Wang et al., 2016), grape (*V. vinifera*) (Zheng et al., 2015), and leafy spurge (*Euphorbia esula*) (Chao et al., 2017). In addition, besides changes of GAs content, we also found that the content of GA_3_ was higher than the other two GAs, indicating an important role GA_3_ plays in dormancy transition of *M. wufengensis*.

Many DEGs were significantly annotated to hormones during dormancy transition based on KEGG annotation. Therefore, the effect of hormones on dormancy was focussed on. In *M. wufengensis*, the ABA content decreased during dormancy release but increased rapidly from 20D_2_ and 21J_2_, and GA_3_ content steadily increased after dormancy release, similar those observed in other species, which suggests a relationship between content of ABA and GA_3_ and the depth of ED. Based on our records, the average temperature during this stage was –4.4°C. Therefore, we hypothesised that a protective strategy to cope with low temperatures may exist in *M. wufengensis*. In addition, a homeostatic network of various hormones is thought to be at the centre of dormancy transition (Shu et al., 2013; Zhang et al., 2018). In *C. praecox*, the ratio of ABA/GA_3_ increases with dormancy breaking (induced by chilling) and decreases during dormancy release in *P. mume* (Wen et al., 2016; Li et al., 2020). Thus, these results are consistent with those of other studies.

Studies of exogenous hormone application have shown that exogenous ABA application can effectively promote dormancy establishment and maintain ED (Zheng et al., 2015; Li and Dami, 2016). Based on previous studies, inhibitory effect of exogenous ABA does not always work all the time and depends on status of buds. In pear (*Pyrus fauriei*), ABA inhibitory effect on bud break could be affected by chilling accumulation (Tamura et al., 2002) and in grape (*V. vinifera*), ABA application to buds which are released from dormancy did not reduce BBP (Zheng et al., 2015). Similar results were found in the present study as 100 μM ABA delayed dormancy release before ECD, whereas 200 and 300 μM ABA could not effectively inhibit BBP. On the one hand, inhibition was not dependent on concentration, which is not consistent with the results observed in grape whose BBP is more efficiently inhibited by high concentration of ABA (Zheng et al., 2015). On the other hand, high concentration of ABA could not suppress bud break in *M. wufengensis* during dormancy release, which is different from results in pear whose BBP with 100, 200, and 300 μM ABA was similar (Li et al., 2018). Compared to 100 μM whose concentration is relatively low, we infer that 200 or 300 μM ABA may damage and then stimulus the defence system of buds in *M. wufengensis*, so higher concentration of ABA treatment has not depressed bud break percentage. These results suggest that a non-ABA-regulated controlling mechanism of dormancy may exist in *M. wufengensis*, and the mechanism needs further study. However, as our experiment was conducted in a climate chamber after buds were cut from trees, whether similar results can be observed in field experiments remains to be investigated.

In addition to physiological data about hormones, we further analysed the data at the molecular level. The content of ABA in plants is not dominated by a single factor but by a balance of biosynthesis and metabolism, and function through various signalling pathways. Therefore, we further analysed the changes in ABA content during dormancy by evaluating ABA biosynthesis, metabolism, and signalling. *NCED* and *CYP707A* are two of the main genes involved in ABA biosynthesis and metabolism, respectively. Overexpression of NCED promotes seed dormancy and delays germination in tobacco (*Nicotiana plumbaginifolia*) (Qin and Zeevaart, 2002). Li et al. (2018) found that *PpNCED-2* and *PpNCED-3* are highly expressed during ED and decrease rapidly during dormancy release. In this transcriptome data, we identified one *NCED* DEG and named the gene *MwNCED-3*. The expression pattern was similar to *PpNCED-3* and consistent with the decrease in ABA content during ED released.

Furthermore, *CYP707A* is known to be highly expressed during dormancy release in peach and pear (Wang et al., 2016; Tuan et al., 2017). Recent studies have shown that *CYP707A* is involved in dormancy release regulation. In potato, ABA content changes are correlated with *NCED* and *CYP707A* gene families and associated with dormancy release in tubers (Destefano-Beltrán et al., 2006). In the present study, the expression of *MwCYP707A-1-2* was downregulated during dormancy release and immediately upregulated during ECD (Figure 6B). This result is not consistent with those of the abovementioned research in peach or pear. Based on these results, we infer that *MwCYP707A-1-2* may be a key gene involved in ED release, and the role of *MwCYP707A-1-2* in bud dormancy of *M. wufengensis* needs to be further studied.

ABA signal transduction is also involved in dormancy regulation and bud dormancy. In pear, Bai et al. (2013) found that *PpPP2Cs* were upregulated while *PpSNRK2s* were downregulated after dormancy release. Similar results were observed by Li et al. (2018) who found the expression of *PpPYLs*, *PpSNRK2s*, and *PpABIs* to be upregulated from PD to ED, but the expression of *PpPP2Cs* was low during ED and increased with the decrease of ABA content during dormancy release. The expression of *VvPP2Cs* higher at PD than at ED (Tuan et al., 2017). From our results, genes associated with ABA signalling showed different expression patterns during dormancy transition. *MwPP2C-6/8/51*, *MwPYL-1/3*, and *MwSNRK2-10* showed a relatively high expression in ED and ED release, while the expression of *MwPP2C-16/24* and *MwABI-5/2-2* in ECD was higher than that in ED. However, the consistency between the trends of their expression and dormancy transition was not significant and their functions need to be further studied.

In addition, ABA could not only influence the expression of ABA-related genes, but also involve in regulation of many dormancy-related genes. In the present study, these dormancy-related genes such as *CYCD*, *PKL* and *CALS1* were differentially affected by ABA: *MwCYCD-3* and *MwCALS-1-1/2* were promoted by ABA and *MwPKL* was depressed by ABA which indicated that a similar ABA-centred model of *H. Aspen* exists in dormancy release *M. wufengensis*. In addition, functions of the dormancy-related genes need to be further study.

### Cold Tolerance and Bud Dormancy in Winter

**FIGURE 10:**
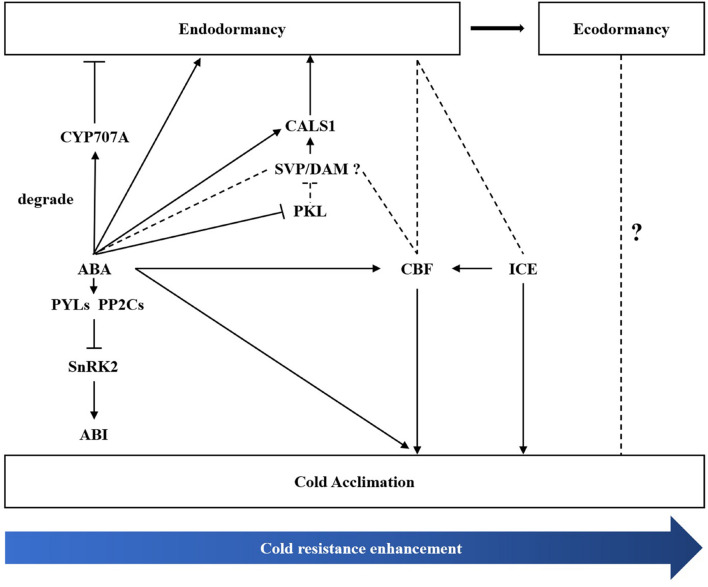
A hypothesis of molecular regulation model between bud dormancy and cold acclimation during winter in *Magnolia wufengensis*.

Bud dormancy, an important biological process for plants to survive the winter, is strongly related to cold hardiness enhancement in winter. Li et al. (2003) found that cold tolerance is enhanced before dormancy development in silver birch (*B. pendula*). In the winter, perennial plants tend to enhance cold tolerance through natural cold acclimation to survive under long-term cold conditions (Uemura et al., 1995; Lee and Thomashow, 2012). The CBF-dependent signalling pathway is an important cold signalling pathway in plants. CBFs/DREBs bind to cis-elements of cold resistance genes and activate their expression, thus improving cold resistance in plants (Park et al., 2015; Ding et al., 2020). ICE1, a positive regulator of cold response, can activate the expression of CBF (Chinnusamy, 2003; Lee et al., 2005). *DAM/SVP* can regulate the bud dormancy cycle in perennial trees (Falavigna et al., 2019). Recently, the relationship between CBF and DAM co-regulating dormancy has been widely studied in many species (Yang et al., 2021). In *P. mume*, *PmDAM6* and *PmCBFs* mainly respond to chilling temperature (below 20°C) and freezing cold (0°C), respectively. Additionally, eight *PmCBFs* were upregulated under the stimulus of a cold signal, which then induced the expression of all six *DAM* genes during dormancy development (Zhang et al., 2018; Zhao et al., 2018a). In the *Populus* hybrid, *PtCBF1* and *PtDAM1* induction was found to be related to ED development (Boldizsár et al., 2021). In pear, Niu et al. (2015) found that *PpCBF* can induce the expression of *PpDAM* and *PpDAM* and subsequently inhibit *PpFT*, which then stimulates growth cessation and promote dormancy maintenance. In the present study, we identified one DREB1B/CBF1 gene (TRINITY_DN8378_c0_g1) and two *ICE1* genes (TRINITY_DN20323_c1_g1 and TRINITY_DN20323_c0_g2). *MwCBF-1* was highly expressed during overwintering, so we suspected that *MwCBF-1* was a positive regulator for cold resistance in *M. wufengensis*. Before dormancy release, *MwCBF-1* achieved a peak at 20N_2_, while the temperature was still above 0°C (Supplementary Figure 7). In the coldest two months, which included D_1_, D_2_, J_1_, and J_2_, *MwCBF-1* decreased but maintained a high expression to cope with cold stress when dormancy was released (Figure 8). Furthermore, the expression of *MwCBF-1* can be efficiently induced by exogenous ABA application. We suspected that *M. wufengensis* can efficiently enhance cold resistance during the dormancy release phase to survive the winter, so it is important to maintain ED and extend the time of the dormancy release phase. Above all, a hypothesis of molecular model for ABA and its biosynthesis, metabolism and signalling pathway; cold tolerance and acclimation; and dormancy during overwintering was proposed (Figure 10). In addition, the role that ECD plays in cold tolerance enhancement during overwintering needs to be further study.

## Conclusion

Overall, this study provides fundamental insights into the bud dormancy cycle and CR in two Magnoliaceae plants and we hypothesised that *M. wufengensis* and *M. denudata* are both sensitive to low temperature and short day based on meteorological data. The content of ABA and GA_3_, and the ABA/GA_3_ ratio significantly changed during dormancy release and ECD. A *M. wufengensis* dataset containing 187,406 unigenes was constructed to observe the dynamic changes in gene expression under different dormancy phases using RNA-seq. Comparison with ED led to the identification of 16,240 and 43,993 DEGs during ED release (19D_1_) and ECD (19D_2_), respectively. Among the DEGs, many key genes and metabolic pathways, especially those of plant hormones, were identified using KEGG and GO analyses. Based on heatmap analysis of plant hormone transduction, we found that auxin- and ABA-related genes showed high expression in ED. Thus, auxin and ABA may regulate dormancy transition in *M. wufengensis*. Application of 100 μM of exogenous ABA before dormancy release could effectively maintain dormancy. Seventeen DEGs involved in ABA biosynthesis, metabolism, and signal transduction were identified based on RNA-seq data. We conducted qPCR on the 17 ABA-related DEGs and found that *MwCYP707A-1-2* may be involved in dormancy regulation. Besides, *MwCBF-1* was highly expressed during dormancy release, suggesting a relationship between cold tolerance and bud dormancy. Thus, our findings shed light on the mechanism underlying dormancy release and further our understanding of overwintering from bud dormancy in *M. wufengensis*.

## Data Availability Statement

The original contributions presented in the study are publicly available. This data can be found here: National Center for Biotechnology Information (NCBI) BioProject database under accession number PRJNA695868 (https://www.ncbi.nlm.nih.gov/bioproject/PRJNA695868).

## Author Contributions

KW performed most of the experiment, analysed the data, and drafted the manuscript. KW and XD designed the experiment, did the bioinformatics analysis, and contributed to the writing of this article. ZZ and ZS provided the experimental materials and participated in data analysis. YZ involved in conducting experiments. HL analysed the data. ZJ and LM initiated and supervised the study. All authors contributed to the article and approved the submitted version.

## Conflict of Interest

The authors declare that the research was conducted in the absence of any commercial or financial relationships that could be construed as a potential conflict of interest.

## Publisher’s Note

All claims expressed in this article are solely those of the authors and do not necessarily represent those of their affiliated organizations, or those of the publisher, the editors and the reviewers. Any product that may be evaluated in this article, or claim that may be made by its manufacturer, is not guaranteed or endorsed by the publisher.

## Footnotes

1
https://github.com/jstjohn/SeqPrep

2
https://github.com/najoshi/sickle

3
https://github.com/trinityrnaseq/trinityrnaseq

4
https://www.blast2go.com/

5
https://www.genome.jp/kegg/

6
https://github.com/deweylab/RSEM

7
https://github.com/tanghaibao/Goatools

8
http://kobas.cbi.pku.edu.cn/home.do

9
https://bioinfogp.cnb.csic.es/tools/venny/index.html

## References

[B1] Anderson J. V. Horvath D. P. Chao W. S. Foley M. E. (2010). “Bud dormancy in perennial plants: a mechanism for survival,” in *Dormancy and Resistance in Harsh Environments. Topics in Current Genetics*, eds Lubzens E. Cerda J. Clark M. (Berlin, Heidelberg: Springer), 69–90.

[B2] Arora R. Rowland L. J. Tanino K. (2003). Induction and release of bud dormancy in woody perennials: a science comes of age. *Hortscience* 38 911–921. 10.21273/HORTSCI.38.5.911

[B3] Atkinson C. Brennan R. M. Jones H. (2013). Declining chilling and its impact on temperate perennial crops. *Environ. Exper. Bot.* 91 48–62. 10.1016/j.envexpbot.2013.02.004

[B4] Bai S. Niu Q. Teng Y. (2016). Advances in the research of the regulatory mechanism of endodormancyin pear (in chinese). *J. Fruit Sci.* 33 1–9.

[B5] Bai S. Saito T. Sakamoto D. Ito A. Fujii H. Moriguchi T. (2013). Transcriptome analysis of japanese pear (pyrus pyrifolia nakai) flower buds transitioning through endodormancy. *Plant Cell Physiol.* 54 1132–1151. 10.1093/pcp/pct067 23624675

[B6] Boldizsár Á Soltész A. Tanino K. Kalapos B. Marozsán-Tóth Z. Monostori I. (2021). Elucidation of molecular and hormonal background of early growth cessation and endodormancy induction in two contrasting populus hybrid cultivars. *BMC Plant Biol.* 21:111. 10.1186/s12870-021-02828-7 33627081 PMC7905644

[B7] Chao W. S. Doğramacı M. Horvath D. P. Anderson J. V. Foley M. E. (2017). Comparison of phytohormone levels and transcript profiles during seasonal dormancy transitions in underground adventitious buds of leafy spurge. *Plant Mol. Biol.* 94 281–302. 10.1007/s11103-017-0607-7 28365837

[B8] Chinnusamy V. (2003). Ice1: a regulator of cold-induced transcriptome and freezing tolerance in arabidopsis. *Genes Dev.* 17 1043–1054. 10.1101/gad.1077503 12672693 PMC196034

[B9] Chinnusamy V. Zhu J. Zhu J. (2007). Cold stress regulation of gene expression in plants. *Trends Plant Sci.* 12 444–451. 10.1016/j.tplants.2007.07.002 17855156

[B10] Conesa A. Götz S. García-Gómez J. M. Terol J. Talón M. Robles M. (2005). Blast2go: a universal tool for annotation, visualization and analysis in functional genomics research. *Bioinformatics* 21 3674–3676. 10.1093/bioinformatics/bti610 16081474

[B11] Considine M. J. Considine J. A. (2016). On the language and physiology of dormancy and quiescence in plants. *J. Exper. Bot.* 67 3189–3203. 10.1093/jxb/erw138 27053719

[B12] Cooke J. E. K. Eriksson M. E. Junttila O. (2012). The dynamic nature of bud dormancy in trees: environmental control and molecular mechanisms. *Plant Cell Environ.* 35 1707–1728. 22670814 10.1111/j.1365-3040.2012.02552.x

[B13] Cutler A. J. Krochko J. E. (1999). Formation and breakdown of aba. *Trends Plant Sci.* 4 472–478. 10.1016/S1360-1385(99)01497-1 10562731

[B14] Deng S. Ma J. Zhang L. Chen F. Sang Z. Jia Z. (2019). De novo transcriptome sequencing and gene expression profiling of magnolia wufengensis in response to cold stress. *BMC Plant Biol.* 19:321. 10.1186/s12870-019-1933-5 31319815 PMC6637634

[B15] Destefano-Beltrán L. Knauber D. Huckle L. Suttle J. C. (2006). Effects of postharvest storage and dormancy status on aba content, metabolism, and expression of genes involved in aba biosynthesis and metabolism in potato tuber tissues. *Plant Mol. Biol.* 61 687–697. 10.1007/s11103-006-0042-7 16897484

[B16] Ding Y. Shi Y. Yang S. (2020). Molecular regulation of plant responses to environmental temperatures. *Mol. Plant* 13 544–564. 10.1016/j.molp.2020.02.004 32068158

[B17] Duan X. Cai C. Yang Y. Chen F. Sang Z. Ma L. (2019). Fall ethephon application enhances the freezing tolerance of magnolia wufengensis during overwintering. *Forests* 10:868. 10.3390/f10100868

[B18] Erez A. Fishman S. Linsley-Noakes G. C. Allan P. (1990). *The dynamic model for rest completion in peach buds.* Leuven, Belgium: International Society for Horticultural Science (ISHS).

[B19] Ernst J. Bar-Joseph Z. (2006). Stem: a tool for the analysis of short time series gene expression data. *BMC Bioinformatics* 7:191. 10.1186/1471-2105-7-191 16597342 PMC1456994

[B20] Falavigna V. Costes E. Andrés F. (2019). I want to (bud) break free: the potential role of dam and svp-like genes in regulating dormancy cycle in temperate fruit trees. *Front. Plant Sci.* 9:1990. 10.3389/fpls.2018.01990 30687377 PMC6335348

[B21] Fishman S. Erez A. Couvillon G. A. (1987a). The temperature dependence of dormancy breaking in plants: mathematical analysis of a two-step model involving a cooperative transition. *J. Theor. Biol.* 124 473–483. 10.1016/S0022-5193(87)80221-7

[B22] Fishman S. Erez A. Couvillon G. A. (1987b). The temperature dependence of dormancy breaking in plants: computer simulation of processes studied under controlled temperatures. *J. Theor. Biol.* 126 309–321. 10.1016/S0022-5193(87)80237-0

[B23] Gao Y. Yang Q. Yan X. Wu X. Yang F. Li J. (2021). High-quality genome assembly of ‘cuiguan’ pear (pyrus pyrifolia) as a reference genome for identifying regulatory genes and epigenetic modifications responsible for bud dormancy. *Horticulture Research* 8:197. 10.1038/s41438-021-00632-w 34465760 PMC8408243

[B24] Grabherr M. G. Haas B. J. Yassour M. Levin J. Z. Thompson D. A. Amit I. (2011). Full-length transcriptome assembly from rna-seq data without a reference genome. *Nat. Biotechnol.* 29 644–652. 10.1038/nbt.1883 21572440 PMC3571712

[B25] Guo X. Liu D. Chong K. (2018). Cold signaling in plants: insights into mechanisms and regulation. *J. Integ. Plant Biol.* 60 745–756. 10.1111/jipb.12706 30094919

[B26] Hao X. Yang Y. Yue C. Wang L. Horvath D. P. Wang X. (2017). Comprehensive transcriptome analyses reveal differential gene expression profiles of camellia sinensis axillary buds at para-, endo-, ecodormancy, and bud flush stages. *Front. Plant Sci.* 8:553. 10.3389/fpls.2017.00553 28458678 PMC5394108

[B27] Heide O. M. (2008). Interaction of photoperiod and temperature in the control of growth and dormancy of prunus species. *Sci. Horticult.* 115 309–314. 10.1016/j.scienta.2007.10.005

[B28] Horvath D. P. Anderson J. V. Chao W. S. Foley M. E. (2003). Knowing when to grow: signals regulating bud dormancy. *Trends Plant Sci.* 8 534–540. 10.1016/j.tplants.2003.09.013 14607098

[B29] Hubbard K. E. Nishimura N. Hitomi K. Getzoff E. D. Schroeder J. I. (2010). Early abscisic acid signal transduction mechanisms: newly discovered components and newly emerging questions. *Genes Dev.* 24 1695–1708. 10.1101/gad.1953910 20713515 PMC2922499

[B30] Ito A. Tuan P. A. Saito T. Bai S. Kita M. Moriguchi T. (2021). Changes in phytohormone content and associated gene expression throughout the stages of pear (pyrus pyrifolia nakai) dormancy. *Tree Physiol.* 41 529–543. 10.1093/treephys/tpz101 31595966

[B31] Lang G. A. Early J. D. Darnell R. Martin G. C. (1987). Endo-, para-, and eco-dormancy: physiological terminology and classification for dormancy research. *Hortscience* 22 371–377.

[B32] Lee B. Henderson D. A. Zhu J. (2005). The arabidopsis cold-responsive transcriptome and its regulation by ice1. *Plant Cell* 17 3155–3175. 10.1105/tpc.105.035568 16214899 PMC1276035

[B33] Lee C. M. Thomashow M. F. (2012). Photoperiodic regulation of the c-repeat binding factor (cbf) cold acclimation pathway and freezing tolerance in arabidopsis thaliana. *Proc. Natl. Acad. Sci.* 109 15054–15059. 10.1073/pnas.1211295109 22927419 PMC3443188

[B34] Li B. Dewey C. N. (2011). Rsem: accurate transcript quantification from rna-seq data with or without a reference genome. *BMC Bioinform.* 12:323. 10.1186/1471-2105-12-323 21816040 PMC3163565

[B35] Li C. Junttila O. Ernstsen A. Heino P. Palva E. T. (2003). Photoperiodic control of growth, cold acclimation and dormancy development in silver birch (betula pendula) ecotypes. *Physiol. Plantarum* 117 206–212. 10.1034/j.1399-3054.2003.00002.x 11841302

[B36] Li J. Xu Y. Niu Q. He L. Teng Y. Bai S. (2018). Abscisic acid (aba) promotes the induction and maintenance of pear (pyrus pyrifolia white pear group) flower bud endodormancy. *Int. J. Mol. Sci.* 19:310. 10.3390/ijms19010310 29361708 PMC5796254

[B37] Li J. Yan X. Yang Q. Ma Y. Yang B. Tian J. (2019). Ppcbfs selectively regulate ppdams and contribute to the pear bud endodormancy process. *Plant Mol. Biol.* 99 575–586. 10.1007/s11103-019-00837-7 30747337

[B38] Li S. Dami I. E. (2016). Responses of vitis vinifera ‘pinot gris’ grapevines to exogenous abscisic acid (aba): i. Yield, fruit quality, dormancy, and freezing tolerance. *J. Plant Growth Regul.* 35 245–255. 10.1007/s00344-015-9529-2

[B39] Li Z. Liu N. Zhang W. Wu C. Jiang Y. Ma J. (2020). Integrated transcriptome and proteome analysis provides insight into chilling-induced dormancy breaking in chimonanthus praecox. *Horticult. Res.* 7:198. 10.1038/s41438-020-00421-x 33328461 PMC7704649

[B40] Liu G. Li W. Zheng P. Xu T. Chen L. Liu D. (2012). Transcriptomic analysis of ‘suli’ pear (pyrus pyrifolia white pear group) buds during the dormancy by rna-seq. *BMC Genom.* 13:700. 10.1186/1471-2164-13-700 23234335 PMC3562153

[B41] Liu J. Sherif S. M. (2019). Hormonal orchestration of bud dormancy cycle in deciduous woody perennials. *Front. Plant Sci.* 10:1136. 10.3389/fpls.2019.01136 31620159 PMC6759871

[B42] Love M. Huber W. Anders S. (2014). Moderated estimation of fold change and dispersion for rna-seq data with deseq2. *Genome Biol.* 15:550. 10.1186/s13059-014-0550-8 25516281 PMC4302049

[B43] Ma L. Y. Wang L. R. He S. C. Liu X. Wang X. Q. (2006). A new species of magnolia (magnoliaceae) from hubei, china. *Bull. Bot. Res.* 26 4–7.

[B44] Maurya J. P. Bhalerao R. P. (2017). Photoperiod- and temperature-mediated control of growth cessation and dormancy in trees: a molecular perspective. *Ann. Bot.* 120 351–360. 10.1093/aob/mcx061 28605491 PMC5591416

[B45] Niu Q. Li J. Cai D. Qian M. Jia H. Bai S. (2015). Dormancy-associated mads-box genes and micrornas jointly control dormancy transition in pear (pyrus pyrifolia white pear group) flower bud. *J. Exper. Bot.* 67 239–257. 10.1093/jxb/erv454 26466664 PMC4682432

[B46] Ogata H. Goto S. Sato K. Fujibuchi W. Bono H. Kanehisa M. (1999). Kegg: kyoto encyclopedia of genes and genomes. *Nucleic Acids Res.* 27 29–34. 10.1093/nar/27.1.29 9847135 PMC148090

[B47] Park S. Lee C. Doherty C. J. Gilmour S. J. Kim Y. Thomashow M. F. (2015). Regulation of the arabidopsis cbf regulon by a complex low-temperature regulatory network. *Plant J.* 82 193–207. 10.1111/tpj.12796 25736223

[B48] Qin X. Zeevaart J. A. D. (2002). Overexpression of a 9-cis-epoxycarotenoid dioxygenase gene in nicotiana plumbaginifolia increases abscisic acid and phaseic acid levels and enhances drought tolerance. *Plant Physiol.* 128:544. 10.1104/pp.010663 11842158 PMC148917

[B49] Richardson E. A. (1974). A model for estimating the completion of rest for ‘redhaven’ and ‘elberta’ peach trees. *Hortscience* 9 331–332.

[B50] Rinne P. Saarelainen A. Junttila O. (1994). Growth cessation and bud dormancy in relation to aba level in seedlings and coppice shoots of betula pubescens as affected by a short photoperiod, water stress and chilling. *Physiol. Plantarum* 90 451–458. 10.1111/j.1399-3054.1994.tb08801.x

[B51] Rinne P. X. Ivi L. H. Welling A. Vahala J. Ripel L. Ruonala R. (2011). Chilling of dormant buds hyperinduces flowering locus t and recruits ga-inducible 1,3-beta-glucanases to reopen signal conduits and release dormancy in populus. *Plant Cell* 23 130–146. 21282527 10.1105/tpc.110.081307 PMC3051240

[B52] Robinson M. D. McCarthy D. J. Smyth G. K. (2009). Edger: a bioconductor package for differential expression analysis of digital gene expression data. *Bioinformatics* 26 139–140. 10.1093/bioinformatics/btp616 19910308 PMC2796818

[B53] Rohde A. Bhalerao R. P. (2007). Plant dormancy in the perennial context. *Trends Plant Sci.* 12 217–223. 17416545 10.1016/j.tplants.2007.03.012

[B54] Saito S. Hirai N. Matsumoto C. Ohigashi H. Ohta D. Sakata K. (2004). Arabidopsis cyp707as encode (+)-abscisic acid 8’-hydroxylase, a key enzyme in the oxidative catabolism of abscisic acid. *Plant Physiol.* 134 1439–1449. 10.1104/pp.103.037614 15064374 PMC419820

[B55] Saito T. Bai S. Imai T. Ito A. Nakajima I. Moriguchi T. (2015). Histone modification and signalling cascade of the dormancy-associated mads-box gene, ppmads13-1, in japanese pear (pyrus pyrifolia) during endodormancy. *Plant Cell Environ.* 38 1157–1166. 10.1111/pce.12469 25311427

[B56] Shi X. Yin Q. Sang Z. Zhu Z. Jia Z. Ma L. (2021). Prediction of potentially suitable areas for the introduction of magnolia wufengensis under climate change. *Ecol. Indicat.* 127:107762. 10.1016/j.ecolind.2021.107762

[B57] Shu K. Huawei Z. Wang S. Chen M. Wu Y. Tang S. (2013). Abi4 regulates primary seed dormancy by regulating the biogenesis of abscisic acid and gibberellins in arabidopsis. *Plos Genet.* 9:e1003577. 10.1371/journal.pgen.1003577 23818868 PMC3688486

[B58] Singh R. K. Maurya J. P. Azeez A. Miskolczi P. Tylewicz S. Stojkoviè K. (2018). A genetic network mediating the control of bud break in hybrid aspen. *Nat. Commun.* 9:4173. 10.1038/s41467-018-06696-y 30301891 PMC6177393

[B59] Singh R. K. Miskolczi P. Maurya J. P. Bhalerao R. P. (2019). A tree ortholog of short vegetative phase floral repressor mediates photoperiodic control of bud dormancy. *Curr. Biol.* 29 128–133. 10.1016/j.cub.2018.11.006 30554900

[B60] Singh R. K. Svystun T. AlDahmash B. Jönsson A. M. Bhalerao R. P. (2017). Photoperiod- and temperature-mediated control of phenology in trees – a molecular perspective. *New Phytol.* 213 511–524. 10.1111/nph.14346 27901272

[B61] Sonnewald S. Sonnewald U. (2014). Regulation of potato tuber sprouting. *Planta* 239 27–38. 10.1007/s00425-013-1968-z 24100410

[B62] Soon F. Ng L. Zhou X. West G. Kovach A. Tan M. (2011). Molecular mimicry regulates aba signaling by snrk2 kinases and pp2c phosphatases. *Science* 335 85–88. 10.1126/science.1215106 22116026 PMC3584687

[B63] Tamura F. Tanabe K. Itai A. (2002). Regulation of endodormancy in japanese pear. *Acta Horticult.* 587 325–336. 10.17660/ActaHortic.2002.587.44

[B64] Tuan P. A. Bai S. Saito T. Ito A. Moriguchi T. (2017). Dormancy-associated mads-box (dam) and the abscisic acid pathway regulate pear endodormancy through a feedback mechanism. *Plant Cell Physiol.* 58 1378–1390. 10.1093/pcp/pcx074 28586469

[B65] Tylewicz S. Petterle A. Marttila S. Miskolczi P. Azeez A. Singh R. K. (2018). Photoperiodic control of seasonal growth is mediated by aba acting on cell-cell communication. *Science* 360 212–215. 10.1126/science.aan8576 29519919

[B66] Uemura M. Joseph R. A. Steponkus P. L. (1995). Cold acclimation of arabidopsis thaliana (effect on plasma membrane lipid composition and freeze-induced lesions). *Plant Physiol.* 109 15–30. 10.1104/pp.109.1.15 12228580 PMC157560

[B67] Umezawa T. Sugiyama N. Mizoguchi M. Hayashi S. Myouga F. Yamaguchi-Shinozaki K. (2009). Type 2c protein phosphatases directly regulate abscisic acid-activated protein kinases in arabidopsis. *Proc. Natl. Acad. Sci.* 106:17588. 10.1073/pnas.0907095106 19805022 PMC2754379

[B68] Vimont N. Fouché M. Campoy J. A. Tong M. Arkoun M. Yvin J. (2019). From bud formation to flowering: transcriptomic state defines the cherry developmental phases of sweet cherry bud dormancy. *BMC Genom.* 20:97.4. 10.1186/s12864-019-6348-z 31830909 PMC6909552

[B69] Vimont N. Schwarzenberg A. Domijan M. Donkpegan A. S. L. Beauvieux R. le Dantec L. (2021). Fine tuning of hormonal signaling is linked to dormancy status in sweet cherry flower buds. *Tree Physiol.* 41 544–561. 10.1093/treephys/tpaa122 32975290

[B70] Wang D. Gao Z. Du P. Xiao W. Tan Q. Chen X. (2016). Expression of aba metabolism-related genes suggests similarities and differences between seed dormancy and bud dormancy of peach (prunus persica). *Fron. Plant Sci.* 6:1248. 10.3389/fpls.2015.01248 26793222 PMC4707674

[B71] Weinberger J. H. (1950). Chilling requirements of peach varieties. *Proc. Amer. Soc. Hort. Sci.* 56 122–128.

[B72] Weiser C. J. (1970). Cold resistance and injury in woody plants. *Science* 169 1269–1278. 10.1126/science.169.3952.1269 17772511

[B73] Wen L. H. Zhong W. J. Huo X. M. Zhuang W. B. Ni Z. J. Gao Z. H. (2016). Expression analysis of aba- and ga-related genes during four stages of bud dormancy in japanese apricot (prunus mume sieb. Et zucc). *J. Horticult. Sci. Biotechnol.* 91 362–369. 10.1080/14620316.2016.1160546

[B74] Xie C. Mao X. Huang J. Ding Y. Wu J. Dong S. (2011). Kobas 2.0: a web server for annotation and identification of enriched pathways and diseases. *Nucleic Acids Res.* 39 W316–W322. 10.1093/nar/gkr483 21715386 PMC3125809

[B75] Yamane H. Ooka T. Jotatsu H. Hosaka Y. Sasaki R. Tao R. (2011). Expressional regulation of ppdam5 and ppdam6, peach (prunus persica) dormancy-associated mads-box genes, by low temperature and dormancy-breaking reagent treatment. *J. Exper. Bot.* 62 3481–3488. 10.1093/jxb/err028 21378115 PMC3130173

[B76] Yang Q. Gao Y. Wu X. Moriguchi T. Bai S. Teng Y. (2021). Comparative analysis of natural cold acclimation and deacclimation of two magnolia species with different winter hardiness. *Horticult. Res.* 8:575. 10.1038/s41438-021-00575-2 34078882 PMC8172858

[B77] Yang Q. Niu Q. Li J. Zheng X. Ma Y. Bai S. (2018). Pphb22, a member of hd-zip proteins, activates ppdam1 to regulate bud dormancy transition in ‘suli’ pear (pyrus pyrifolia white pear group). *Plant Physiol. Biochem.* 127 355–365. 10.1016/j.plaphy.2018.04.002 29677681

[B78] Yang Q. Niu Q. Tang Y. Ma Y. Yan X. Li J. (2019). Ppygast1 is potentially involved in bud dormancy release by integrating the ga biosynthesis and aba signaling in ‘suli’ pear (pyrus pyrifolia white pear group). *Environ. Exper. Bot.* 162 302–312. 10.1016/j.envexpbot.2019.03.008

[B79] Yang Q. Yang B. Li J. Wang Y. Tao R. Yang F. (2020). Aba-responsive abre-binding factor3 activates dam3 expression to promote bud dormancy in asian pear. *Plant Cell Environ.* 43 1360–1375. 10.1111/pce.13744 32092154

[B80] Yang Y. Jia Z. Chen F. Sang Z. Ma L. (2015). Comparative analysis of natural cold acclimation and deacclimation of two magnolia species with different winter hardiness. *Acta Physiol. Plantarum* 37:129. 10.1007/s11738-015-1883-y

[B81] Yooyongwech S. Sugaya S. Sekozawa Y. Gemma H. (2009). Differential adaptation of high- and low-chill dormant peaches in winter through aquaporin gene expression and soluble sugar content. *Plant Cell Rep.* 28:1709. 10.1007/s00299-009-0770-7 19760270

[B82] Yue C. Cao H. Hao X. Zeng J. Qian W. Guo Y. (2018). Differential expression of gibberellin- and abscisic acid-related genes implies their roles in the bud activity-dormancy transition of tea plants. *Plant Cell Rep.* 37 425–441. 10.1007/s00299-017-2238-5 29214380

[B83] Zhang Z. Zhuo X. Zhao K. Zheng T. Han Y. Yuan C. (2018). Transcriptome profiles reveal the crucial roles of hormone and sugar in the bud dormancy of prunus mume. *Sci. Rep.* 8:5090. 10.1038/s41598-018-23108-9 29572446 PMC5865110

[B84] Zhao J. Li G. Yi G. Wang B. Deng A. Nan T. (2006). Comparison between conventional indirect competitive enzyme-linked immunosorbent assay (icelisa) and simplified icelisa for small molecules. *Anal. Chim. Acta* 571 79–85. 10.1016/j.aca.2006.04.060 17723423

[B85] Zhao K. Zhou Y. Ahmad S. Yong X. Xie X. Han Y. (2018a). Pmcbfs synthetically affect pmdam6 by alternative promoter binding and protein complexes towards the dormancy of bud for prunus mume. *Sci. Rep.* 8:4527. 10.1038/s41598-018-22537-w 29540742 PMC5852209

[B86] Zhao K. Zhou Y. Li Y. Zhuo X. Ahmad S. Han Y. (2018b). Crosstalk of pmcbfs and pmdams based on the changes of phytohormones under seasonal cold stress in the stem of prunus mume. *Int. J. Mol. Sci.* 19:15. 10.3390/ijms19020015 29360732 PMC5855539

[B87] Zheng C. Acheampong A. Shi Z. Mugzech A. Halaly Basha T. Shaya F. (2018). Abscisic acid catabolism enhances dormancy release of grapevine buds. *Plant Cell Environ.* 41:13371. 10.1111/pce.13371 29907961

[B88] Zheng C. Halaly T. Acheampong A. K. Takebayashi Y. Jikumaru Y. Kamiya Y. (2015). Abscisic acid (aba) regulates grape bud dormancy, and dormancy release stimuli may act through modification of aba metabolism. *J. Exper. Bot.* 66 1527–1542. 10.1093/jxb/eru519 25560179 PMC4339608

[B89] Zhuang W. Gao Z. Wang L. Zhong W. Ni Z. Zhang Z. (2013). Comparative proteomic and transcriptomic approaches to address the active role of ga4 in japanese apricot flower bud dormancy release. *J. Exper. Bot.* 64 4953–4966. 10.1093/jxb/ert284 24014872 PMC3830480

