# Supplementary material for: Transcriptomic Analysis Reveals the Positive Role of Abscisic Acid in Endodormancy Maintenance of Leaf Buds of Magnolia wufengensis
Source: Front Plant Sci. 2021 Nov 11;12:742504. doi: 10.3389/fpls.2021.742504 (PMC8632151; doi:10.3389/fpls.2021.742504)
Supplement: Supplementary file 1 [file Data_Sheet_1.pdf]

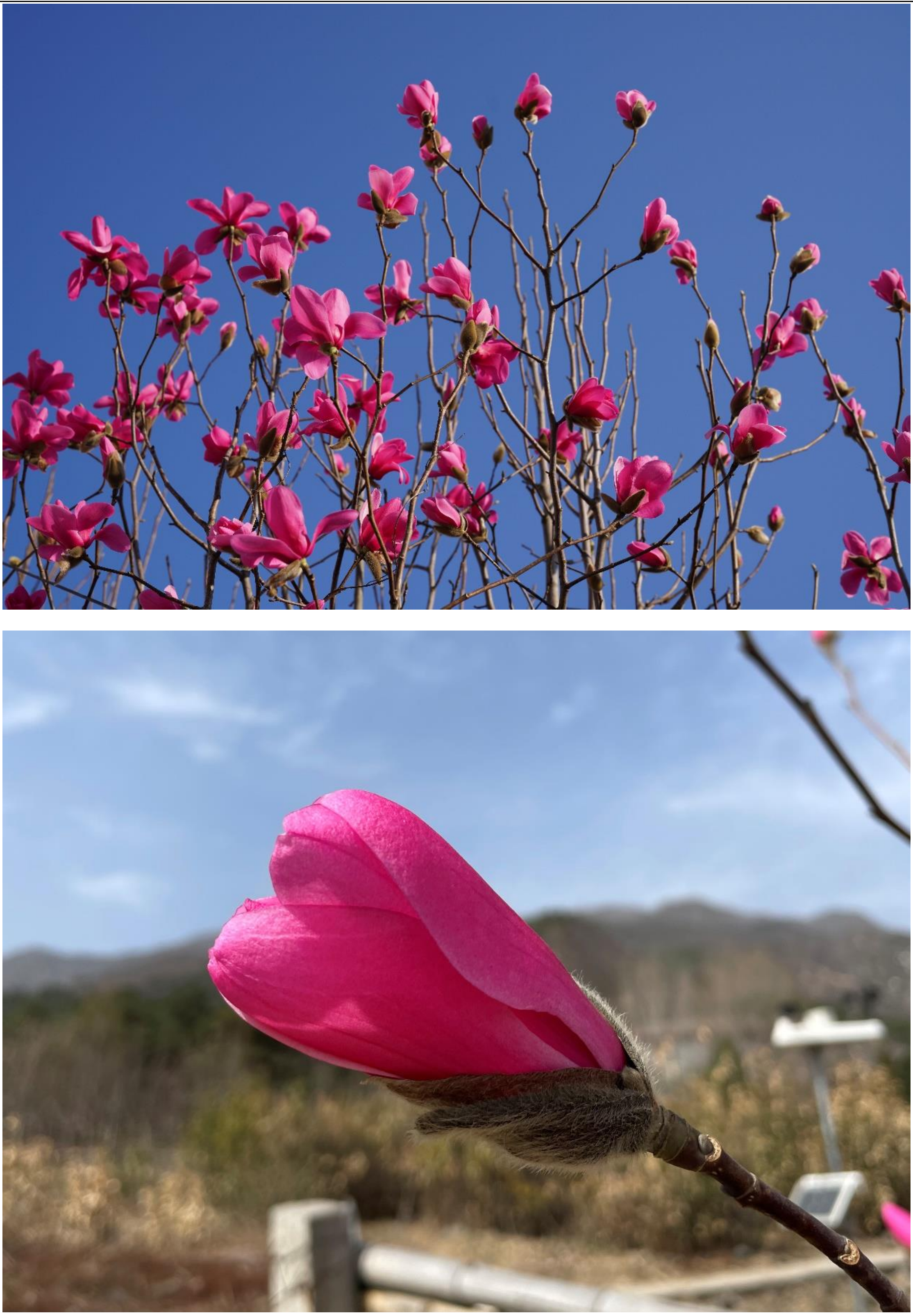

Supplementary Figure 1. Flowers of *Magnolia wufengensis*.

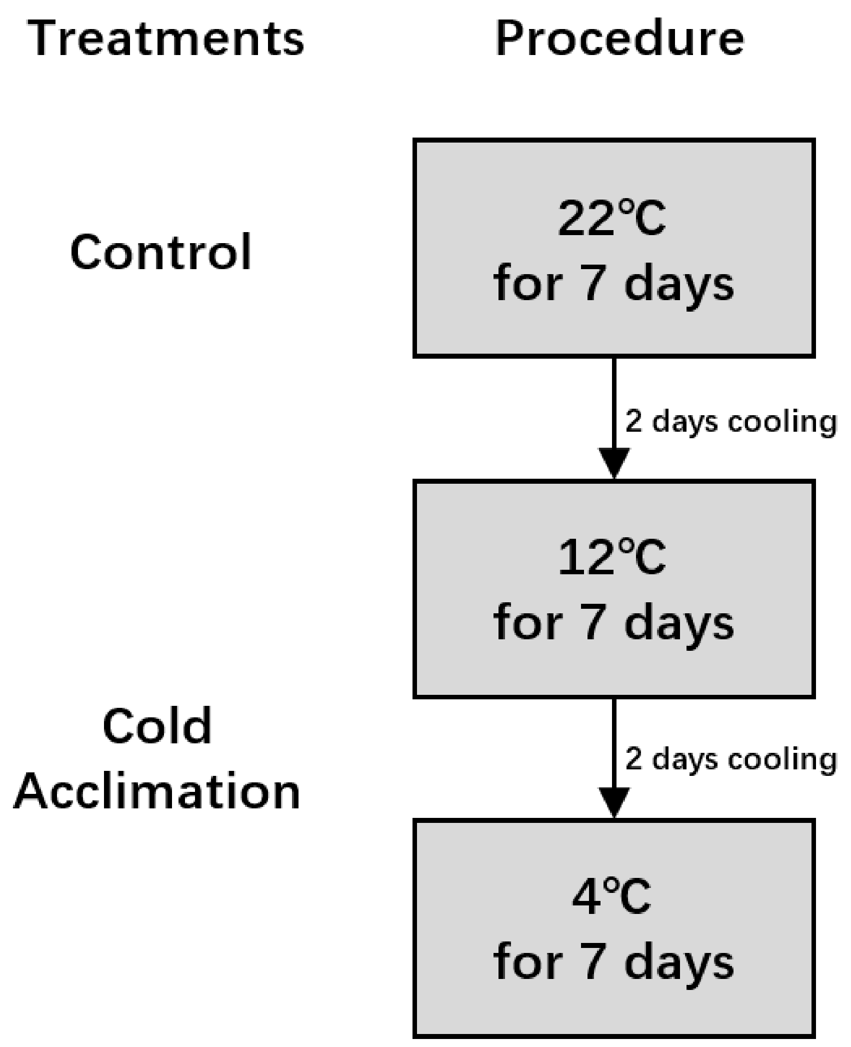

Supplementary Figure 2. Experimental design of cold acclimation in *Magnolia wufengensis*

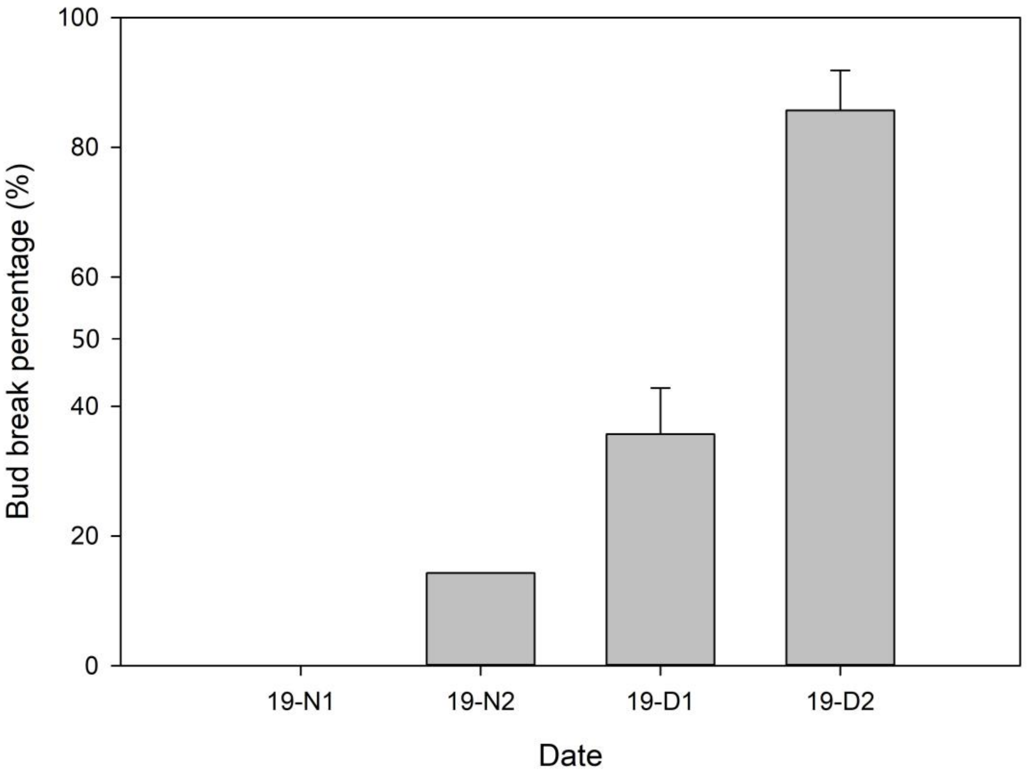

Supplementary Figure 3. Bud break percentage of *Magnolia wufengensis* during 2019–2020 dormancy phase under natural conditions. Bud break percentage of *Magnolia wufengensis* was assessed after 32 days in 2019–2020. Different letters above bars indicate a significant difference among bud break percentage according to ANOVA and LSD test ($p < 0.05$).

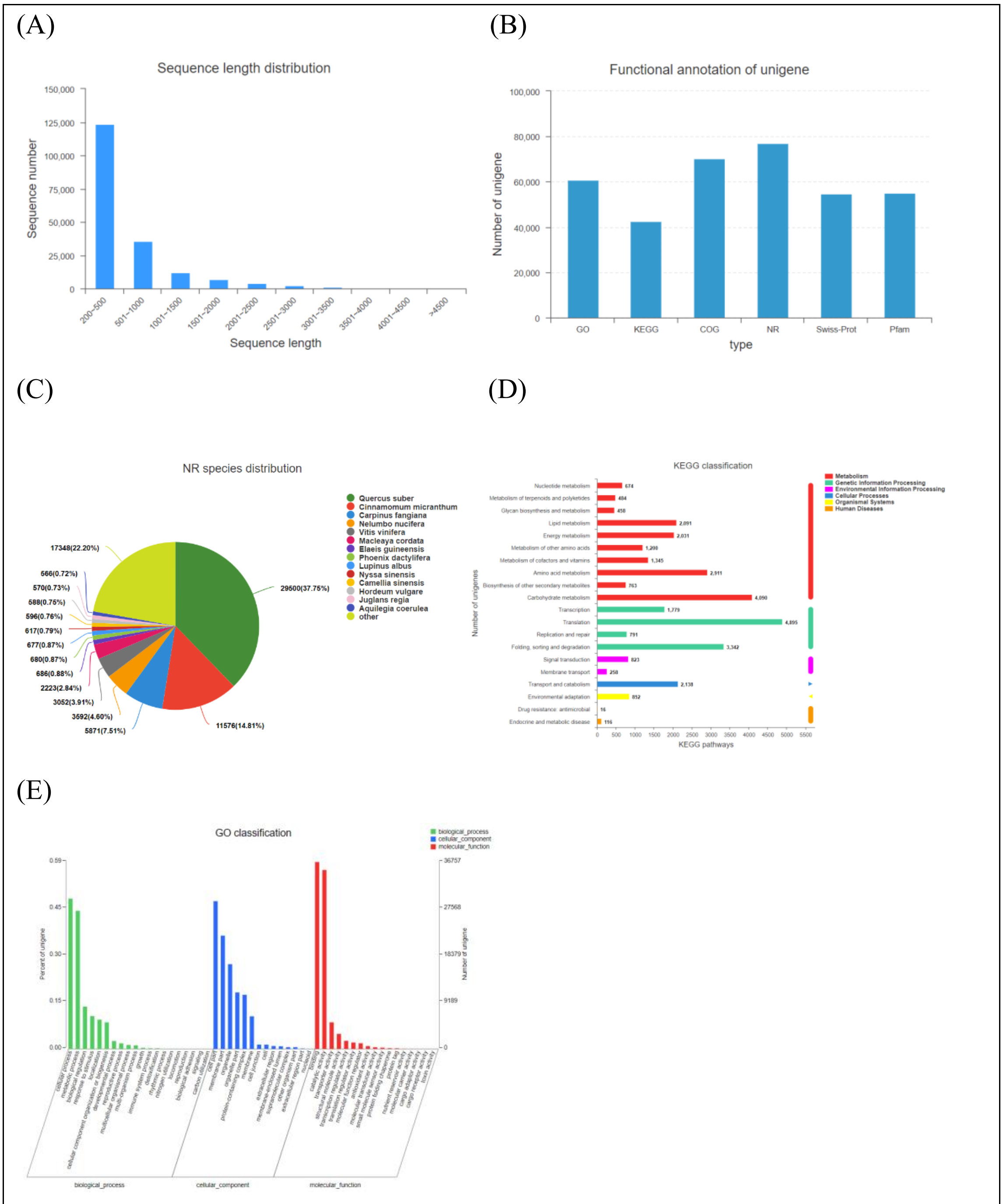

Supplementary Figure 4. Characteristics and annotations of unigenes.

(A) Unigene lengths distribution in *M. wufengensis* **(B)** Functional annotations of unigenes in *Magnolia wufengensis* **(C)** Top 14 Species distribution for each unigene based on NR database **(D)** KEGG annotation of the unigenes **(E)** GO categorization functional annotation of the unigenes

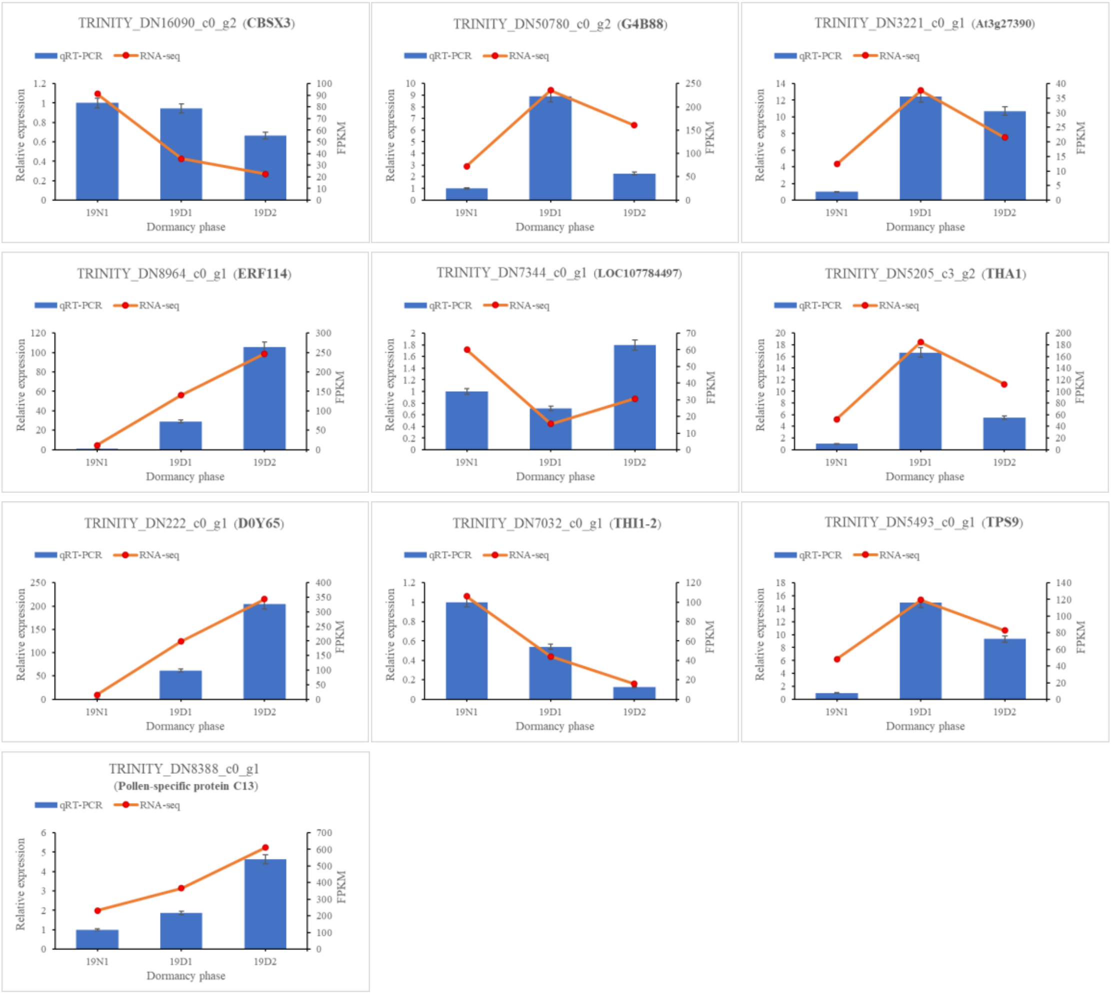

Supplementary Figure 5. The expression of 10 DEGs from endodormancy (19N$_1$), endodormancy release (19D$_1$), ecodormancy (19D$_2$) and comparison between qPCR and RNA-seq. Each experiment was performed with three biological and technical replicates.

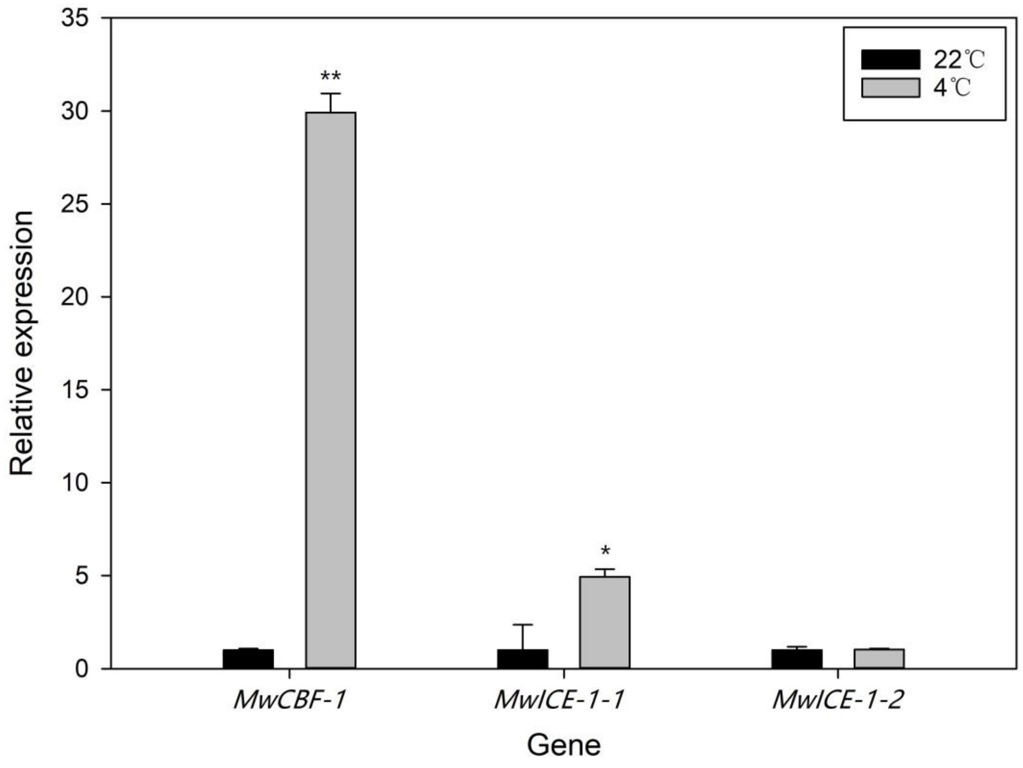

Supplementary Figure 6. Relative expressions of *MwCBF-1*, *MwICE-1-1* and *MwICE-1-2* under cold acclimation. Error bars indicate standard errors from three biological replicates (* $p < 0.05$, $p < 0.01$) while means with different letters had significant differences ($p < 0.05$).**

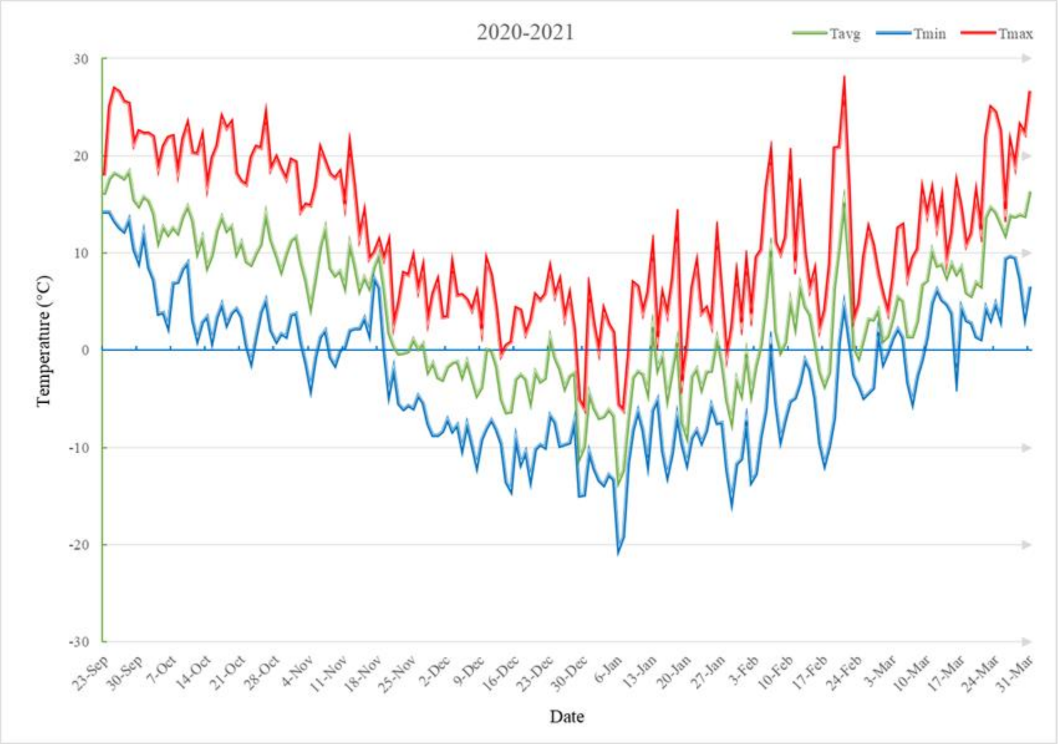

Supplementary Figure 7. Daily average, minimum, and maximum temperatures during 2020–2021.
